# BRK Targets Dok1 for Ubiquitin-Mediated Proteasomal Degradation to Promote Cell Proliferation and Migration

**DOI:** 10.1371/journal.pone.0087684

**Published:** 2014-02-11

**Authors:** Sayem Miah, Raghuveera Kumar Goel, Chenlu Dai, Natasha Kalra, Erika Beaton-Brown, Edward T. Bagu, Keith Bonham, Kiven E. Lukong

**Affiliations:** 1 Department of Biochemistry, College of Medicine, University of Saskatchewan, Saskatoon, Saskatchewan, Canada; 2 Cancer Research Unit, Health Research Division, Saskatchewan Cancer Agency, and Division of Oncology, College of Medicine, University of Saskatchewan, Saskatoon, Saskatchewan, Canada; University of Illinois at Chicago, United States of America

## Abstract

Breast tumor kinase (BRK), also known as protein tyrosine kinase 6 (PTK6), is a non-receptor tyrosine kinase overexpressed in more that 60% of human breast carcinomas. The overexpression of BRK has been shown to sensitize mammary epithelial cells to mitogenic signaling and to promote cell proliferation and tumor formation. The molecular mechanisms of BRK have been unveiled by the identification and characterization of BRK target proteins. Downstream of tyrosine kinases 1 or Dok1 is a scaffolding protein and a substrate of several tyrosine kinases. Herein we show that BRK interacts with and phosphorylates Dok1 specifically on Y362. We demonstrate that this phosphorylation by BRK significantly downregulates Dok1 in a ubiquitin-proteasome-mediated mechanism. Together, these results suggest a novel mechanism of action of BRK in the promotion of tumor formation, which involves the targeting of tumor suppressor Dok1 for degradation through the ubiquitin proteasomal pathway.

## Introduction

Breast tumor kinase (BRK) is a non-receptor tyrosine kinase that was first identified while screening for protein tyrosine kinases in cultured human melanocytes [1] and later in breast tumors [2]. BRK is overexpressed in over 60% of human breast carcinomas, but not in normal mammary glands or benign lesions [3], [4], [5], [6]. Its overexpression has also been observed in other cancers including some metastatic melanomas [7], colon cancers [8], squamous cell carcinomas [9], prostate cancers [10], malignant lymphocytes [11], as well as in high-grade serous carcinomas and ovarian cancer cell lines [12].

BRK belongs to the tyrosine kinase family which includes Frk, Srm and Src42A [13]. The encoded 451 amino acid polypeptide of BRK is composed of a Src homology 3 (SH3) domain, an SH2 domain and a kinase domain with a putative C-terminal regulatory tyrosine and displays a similar architecture to and has 30–40% sequence identity with Src kinases [13]. Unlike Src family kinases, BRK lacks the myristoylated N-terminal consensus sequence required for membrane anchorage and therefore localizes in the nucleus and the cytoplasm. Like Src kinases, BRK is regulated negatively by phosphorylation of the C-terminal tyrosine 447 (which is analogous to the regulatory Y530 of Src) and positively by phosphorylation of tyrosine 342 in the catalytic domain (as Y419 of Src) [14], [15]. Others and we have shown that mutation of tyrosine 447 to phenylalanine significantly enhances the kinase activity of BRK [14], [15], [16].

The cellular roles of BRK in breast cancer have not been fully elucidated; however, overexpression and constitutive activation of BRK in non-transformed human mammary epithelial cells or BRK-negative breast cancer cells induces anchorage-independent growth and increased cell survival, respectively [17], [18]. Several studies have revealed that BRK enhances EGFR tyrosine kinase signaling and positively regulates breast cancer cell growth and migration [17], [19], [20], [21], [22], [23]. In breast carcinomas, expression of BRK was highest in cancers that also expressed in HER2 and HER4 [6], [24]. Although no specific BRK signaling pathway has been delineated, BRK is implicated in several signaling cascades. Consistent with its potential role in tumorigenesis, BRK associates with EGFR, enhancing the mitogenic signals by promoting the recruitment of phosphatidylinositol 3-kinase (PI3K) and activating Akt as well as stimulating cell migration by activating signalling molecules such as Mitogen-activated protein kinase (MAPK) and paxillin [17], [19], [20], [25]. In addition, data from a recent BRK mouse model revealed that BRK promotes increased cell survival, delayed involution, and latent tumor formation by inducing p38-driven pro-survival signaling pathways [26].

More recently, it was demonstrated that depletion of BRK in breast cancer cells impairs the activation of EGFR-regulated signaling molecules [27]. Recent data from our group showed significantly increased MAPK activity, cell proliferation and migration in breast cancer cells stably expressing BRK-Y447F, and decreased migration in breast cancer cells depleted of BRK [28]. These findings as a whole strongly suggest a role for BRK in promoting cell proliferation and migration.

The identification and characterization of an expanding repertoire of BRK interacting proteins and substrates have significantly improved our understanding of the molecular and cellular functions of BRK. We have shown that the BRK substrate Sam68 (Src associated during mitosis, 68 kDa) is an effector of EGF stimulation and that BRK contributes to Sam68 phosphorylation in the EGF-treated breast cancer cells [16], [29]. Other substrates such as paxillin [20], serine/threonine kinase protein kinase B/Akt [23], insulin receptor substrate-4 (IRS-4) [30], signal transducer and activator of transcription 3 (STAT3) [31], STAT5b [32], p190 [20], [25], kinesin-associated protein 3A [33] and polypyrimidine tract-binding (PTB) protein-associated splicing factor (PSF) [34] have also linked BRK to signal transduction. STAT3 for instance is phosphorylated and specifically activated by BRK, resulting in increased cell proliferation [31]. One of STAT3 target gene products is the suppressor of cytokine signaling 3 (SOCS3), which was recently shown to inhibit BRK-induced activation of STAT3 [35]. BRK phosphorylation of paxillin and p190 results in the activation of the small GTPase Rac1 and the induction of cell migration and cell invasion in an EGF-dependent manner [20], [25]. BRK was shown to phosphorylate STAT5b on Y699, which enhanced STAT5b transcriptional activity and suggests that BRK signals downstream to STAT5b to mediate the proliferation of breast cancer cells [32]. We have demonstrated that KAP3A is required by BRK in the induction of cell migration and that the phosphorylation of PSF by BRK results in cell cycle arrest [33], [34]. Furthermore, stimulation of insulin-like growth factor-1 receptor (IGF-1R) in human breast cancer results in the activation of BRK [30]. Heat shock protein 90 (Hsp90) was recently identified as a BRK interacting protein and shown to stabilize BRK in breast cancer cells [36]. BRK has been shown to regulate clathrin-mediated EGFR endocytosis via phosphorylation of ARAP1 (Arf-GAP, Rho-GAP, ankyrin repeat, and pleckstrin homology (PH) domain-containing protein 1) [37] and also to interact with EGFR and inhibit ligand-induced EGFR degradation [38].

Overexpression of BRK has been shown to result in the phosphorylation of numerous unidentified cellular targets [28], [34]. In a recent proteomic study, downstream of tyrosine kinase 1 (Dok1), a tumor suppressor, was identified as a potential substrate of BRK [39]. Therefore, to further understand the cellular roles of BRK, we explored the functional link between BRK and Dok1. Dok1 is a scaffolding protein which mediates protein-protein interactions and has been shown to be phosphorylated by several tyrosine kinases including SRMS, v-Src, c-Abl and p210-Bcr-Abl [40], [41], [42], [43], [44], [45]. Herein we show that BRK interacts with and phosphorylates Dok1 predominantly on Y362, promoting its proteasome-mediated degradation.

## Materials and Methods

### Antibodies and Reagents

The following antibodies were purchased from Santa Cruz Biotechnology (Santa Cruz, CA, USA): anti-BRK (sc-916), anti-tubulin (Sc-9104), anti GFP (Sc-8334) and anti-phosphotyrosine (Sc-508), anti-β-actin (sc-130300). Anti-Dok1 was a gift from Dr. Ryuji Kobayashi (University of Texas, Austin, USA). The anti-Sam68 (AD1) polyclonal antibody has been previously described [29]. Proteasome inhibitors MG132 or Lactacystin were purchased from Calbiochem (MA, USA), cycloheximide from Sigma-Aldrich Corporation (St. Louis, MO) and EGF from Upstate (Lake placid, NY).

### Dok1 expression vectors and mutagenesis

A GFP-Dok1 construct (gift from Dr. Bakary S. Scylla, Lyon, France) was used to generate GFP-Dok1 deletion mutants. Five pairs of primers were used to amplify five Dok1 cDNA variants of progressively differing lengths which were then cloned at the C-terminal of the GFP sequence between the EcoRI and SmaI restriction enzymes sites of the pEGFP-C1 vector backbone: DokΔ1: 5′-AGT GAA TTC GGA CGG AGC AGT GAT GGA A-3′ and 3′- ATT CCC GGG TCA AGT CTC AAC TGC CTG-5′; DokΔ2: 5′-AGT GAA TTC GGA CGG AGC AGT GAT GGA A -3′ and 3′-ATT CCC GGG TCA CTT CCG TTG TAC TCC-5′; DokΔ3: 5′-AGT GAA TTC GGA CGG AGC AGT GAT GGA A-3′ and 3′-ATT CCC GGG TCA CTT GGC CTT CAG CAA-5′; DokΔ4: 5′-AGT GAA TTC GGA CGG AGC AGT GAT GGA A and 3′-ATT CCC GGG TCA CTT CAC CCG AGC TTG-5′; DokΔ5: 5′-AGT GAA TTC GGA CGG AGC AGT GAT GGA A-3′ and 3′-ATT CCC GGG TCA CTT GGG AGC AAG GAG-5′. The Dok1 C-terminal segment extending from the IRS-PTB and spanning 222 amino acids was cloned into EcoRI - NotI sites of the pGEX-5-x-3 vector, using the primers: 5′-ATA GAA TTC CGA CGG AGC AGT GAT GGA A-3′ and 5′-ATA GCG GCC GCT CAG GTA GAG CC-3′. The Dok1 cDNA was cloned at the C-terminal of the mCherry (a generous gift from Dr. Scott Stone, University of Saskatchewan, Saskatoon, SK, Canada) sequence between the BglII and SmaI restriction sites of the pmCherry-C1 vector backbone using the primers: 5′-AAA AGA TCT ATG GAC GGA GCA GTG ATG and 3′-ATT CCC GGG TCA GGT AGA GCC CTC TGA. The composite mcherry-Dok1 cDNA was subcloned between the KpnI and NotI restriction enzymes sites of a pShuttle-CMV plasmid by using a set of primers 5′-AAA GGT ACC GTC GCC ACC ATG GTG AGC AAG GGC GAG and 3′-ATA GCG GCC GCT CAG GTA GAG CC. Site-directed mutations of human Dok1 were introduced using a Quick Change Site-Directed Mutagenesis Kit (Stratagene, La Jolla, CA) according to the manufacturer's instructions. All constructs were verified by sequencing.

### Cell cultures

HEK 293, BT20, MCF-10A, AU565, MDA-MB-231, MDA-MB-435, MDA-MB-468, T47D, HBL100, MCF7 and SKBR3 cells were obtained from the American Type Culture Collection (ATCC, Manassas, VA, USA). The cells were cultured in high glucose (4.5 g/l), Dulbecco's modified Eagle's medium (DMEM) supplemented with 10% bovine calf serum (Thermo scientific, Logan, USA) and containing 4 mM L-glutamine, 100 units/ml penicillin, 100 µg/ml streptomycin (Sigma-Aldrich St. Louis, MO).

### Generation of stable cell lines

The HEK 293 stable cell lines showing constitutive expression of BRK were generated as described previously [28]. Endogenous BRK was stably knocked down in the SKBR3 breast cancer cell line using BRK-specific short-hairpin RNA (shRNA) lentiviral plasmids (Santa Cruz, CA, USA), as recommended by the manufactures. SKBR3 or SKBR3 cells in which BRK was knocked down, were treated with EGF (100 ng/ml) for 5, 10, 15 and 30 minutes while control cells were cultured in the vehicle.

### Preparation of cell lysates

Confluent or subconfluent cells were washed twice with ice-cold PBS. Unless specified otherwise, all procedures were carried out at 4°C (on ice). Cells were lysed using freshly prepared lysis buffer (20 mM Tris ph 7.5, 1% triton, 150 mm Nacl, protease inhibitors: Aprotinin 5 mg/l and PMSF 0.1 mM and centrifuged at 14, 000 rpm for 15 minutes at 4°C. To obtain whole-cell lysates, cells were lysed in SDS sample buffer [50 mM Tris/HCl (pH 6.8), 2% SDS, 0.1% Bromophenol Blue and 10% glycerol].

### Mammalian cell expression and Immunoprecipitation

All transfections were carried out in the HEK293 cell line. Cells, cultured in 6 well plates, were transiently transfected with a total of 2.5 µg of DNA per well using 1% Polyethyleneimine ‘Max’ (PEI) (Polysciences Inc., Warrington, PA, USA) at a DNA to transfection reagent ratio: 1∶6. For each well in the 6-well plate, 2.5 µg of the appropriate DNA was mixed with 107.5 µl of 0.15M sterile NaCl by gently vortexing for 10 seconds. 15 µl of the transfection reagent, PEI, was then added to the mixture followed by another 10 seconds of gentle vortexing. DNA-PEI complex formation was then allowed to take place by incubating the mixture at room temperature for 10 minutes followed by dispensing the mixture dropwise into the wells. The cells were then harvested 24 hours post-transfection.

Whole cell lysates were directly prepared in 2× Laemmli buffer (Sigma). For immunoprecipitation, cells were washed with cold 1× Phosphate-buffered saline (PBS), lysed with freshly prepared lysis buffer (20 mM Tris ph 7.5, 1% triton, 150 mm Nacl, protease inhibitors: Aprotinin 5 mg/l and 0.1 mM PMSF), containing 0.3 mM sodium orthovanadate (Enzo life sciences). Lysates were prepared by incubating the harvested cells in ice-cold lysis buffer for 30 minutes followed by centrifugation for 10 minutes at 14, 000 rpm. Supernatants were collected and transferred into fresh tubes and incubated with 1 µg of the appropriate antibody and maintained on a gyrorotator for 1 hour at 4°C. 20 µl of Protein A beads were then added to the samples and incubated for another 40 minutes on the gyrotator at 4°C. The beads were washed twice with ice-cold lysis buffer and 1× PBS and the immunoprecipitated proteins were resolved via SDS-PAGE.

### Immunoblotting

Proteins derived from either whole cell lysates or derived from immunoprecipitations, were resolved via SDS-PAGE in 10% polyacrylamide gels. The resolved proteins were then transferred onto nitrocellulose membranes (Bio-RAD, Hercules, CA, USA) and immunoblotted with the appropriate antibodies via incubation overnight at 4°C. Polyclonal goat HRP-conjugated secondary antibodies (Bio-Rad Inc., Hercules, CA) against rabbit or mouse were used at a working dilution of 1∶10,000 for membrane incubation at 4°C. Enhanced chemiluminiscence (ECL) (DuPont, Wilmington, DE, USA) was then utilized to detect the immunoreactive proteins on the membranes.

### RT-PCR and qPCR

Total RNA was extracted using the RNeasy mini plus kit (Qiagen, Maryland, USA). Quantity and quality of RNA was determined spectrophotometrically and then 1 µg of the RNA was utilized for cDNA synthesis using the iScript cDNA Synthesis kit (Bio-Rad Inc., Hercules, CA).

Real Time PCR- Quantification of cDNA was performed using a fluorescence based real time detection system (Step One Plus, Applied Biosystems). Using Dok1 primers, PCR was performed in a final volume of 10 µL containing 0.3 µL cDNA, 33 ng of each primer and 5 µL of SsoFast EvaGreen Supermix (Bio-Rad Inc., Hercules, CA). Cycling conditions were: 20 seconds at 95°C followed by 40 cycles of 95°C for 3 seconds, and 58.2°C for 30 seconds. Data was analyzed by the ΔCt method. qPCR was performed using 50 ng of cDNA in 50 µL reaction mixtures containing 0.02 mM deoxynucleoside triphosphate (dNTP) mix (GenScript, NJ, USA), 10× Standard Taq buffer (New England Biolabs, MA, USA), 0.0125 U/µL of Taq DNA polymerase (New England Biolabs, MA, USA), and 0.4 mM of each primer. Primers specific for human Dok1 and reference RPL 13A were the following: Dok-1 forward 5′-CTA CAA CCC TGC CAC TGA TGA CTA-3′ and reverse primer 3′-CTA GAG AGC CCA CAG TCC CAG CTC-5′; RPL13A forward 5′-CAA GGT GTT TGA CGG CAT CC-3′; an reverse primer, 3′ GCT TTC TCT TTC CTC TTC TCC 5′. The cDNAs used in the mixtures were prepared from HEK 293, HEK293-GFP-BRK-WT and HEK 293-BRK-YF. The cycling program used was 95°C for 2 minutes, followed by 35 PCR cycles (95°C for 30 seconds, 60°C for 15 seconds, 72°C for 45 seconds) and a final extension for 5 minutes at 72°C. PCR products were run on 1% agarose gels and visualized by GelRed staining using the AlphaDigiDocTM gel doc (Genetic Technologies, Inc, USA).

### Recombinant GST-fused Protein expression and GST-pull-down assay

GST pull-down assays were performed as previously described [46]. GST-tagged constructs were expressed in E.coli (BL21 strain), cultured in 2XYT media. Protein induction was initiated by the addition of 1 mM IPTG to the bacterial cultures at an optical density of 0.6. Bacterial cells were then lysed by sonication in ice-cold 1×PBS buffer containing protease inhibitors: 1 µg/mL aprotinin, and 0.01% phenylmethanesulfonyl fluoride (PMSF), supplemented with a protease inhibitor cocktail comprising 23 mM AEBSF (4-(2-Aminoethyl) benzenesulfonyl fluoride hydrochloride), 2 mM Bestatin, 100 mM EDTA (Ethylene Di Amine Tetra Acetic acid), E-64 0.3 mM trans-epoxysuccinyl-L-leucylamido-(4-guanidino) butane (E 64), 0.3 mM Pepstatin A, in dimethylsulfoxide (DMSO) (P8465, Sigma-Aldrich Corporation, St. Louis, MO). Lysates were then incubated with the Glutathione sepharose beads (GST, Novagen, CA, USA.). In brief, the pull-down experiments were carried out using GST, GST-BRK-SH3 and GST-BRK-SH2 proteins immobilized on glutathione Sepharose beads, which were incubated with cell lysates, the bound proteins were then resolved by SDS-PAGE as described above.

### *In vitro* Kinase Assay

*In vitro* kinase assays were performed using GST-BRK and a 10 µl volume of substrate (GST-C-terminus Dok1) in a reaction volume of 50 µl comprising 20 µl kinase buffer (25 mM MOPS, pH 7.2, 2.5 mM DTT, 12.5 mM and 5 mM EGTA (Signalchem, Richmond, BC, Canada) with or without 200 µM ATP. The reaction mixture was incubated at 30°C for 30 minutes to complete the kinase reaction and eventually terminated by the addition of 2× laemmli. The samples were then boiled at 100°C and resolved via SDS-PAGE (as described above).

### *In vivo* ubiquitination Assays

GFP-BRK-YF expressing HEK 293 stable cells were transfected with HA-tagged ubiquitin and/or Dok1 expressing adenovectors and the cells treated with 10 µM MG132. The cell lysates were incubated with primary rabbit anti-Dok1 antibody, followed by protein A agarose conjugation and immunoblotting with anti-HA antibody to detect ubiquitinated Dok1.

### Cell migration (Wound healing) Assay

Cells were seeded into 6 well plates at a density of 1×10^6^ cells/well and cultured to 80–90% confluence in complete media as previously described. A 1000 µl sterile pipette tip was used to introduce a longitudinal scratch along the diameter of each well through the monolayer of the confluent cells. The media and cell debris were aspirated away and replaced with a fresh culture media. In order to evaluate cell migration, images of the wells were captured at 0 and 24 hours post-wounding using the Olympus 1X51 inverted microscope (Olympus America, Center Valley, PA)

### Statistical Analysis

One-way ANOVA followed by a post hoc Newman-Keuls test was used for multiple comparisons using GraphPad Prism version 5.04 for Windows, GraphPad Software, San Diego California USA, www.graphpad.com. The results are presented as the mean ± SD, n≥3 unless otherwise stated. P≤0.05 was considered statistically significant.

## Results

### Dok1 is a substrate of BRK

In a recent report it was suggested that Dok1 was a potential substrate of BRK [39], as such we therefore we investigated whether Dok1 was an endogenous target of BRK. In the present study we used a mutant BRK-Y447F that was previously reported to have a higher enzymatic activity than BRK-WT or KM (Figure S1 in File S1) [29].

**Figure 1:**
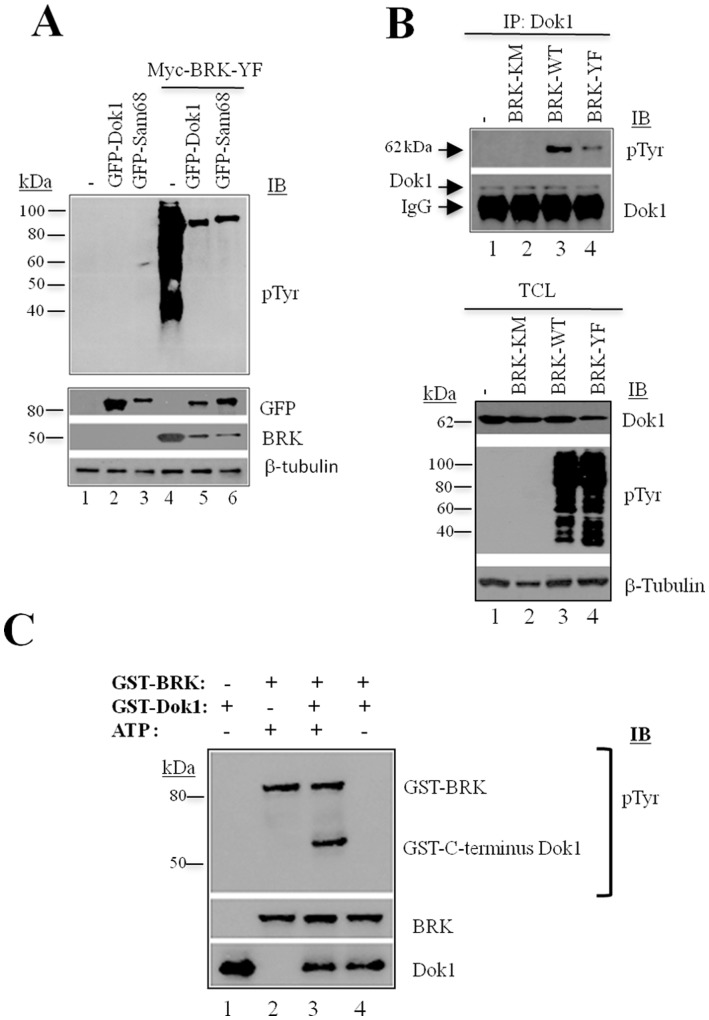
Dok1 is a direct substrate of BRK. (A) HEK 293 cells were transiently transfected with empty control vector (−) or GFP-Dok1, GFP-Sam68, Myc-BRK or co-transfected with Myc-BRK+GFP-Sam68 and Myc-BRK+GFP-Dok1. Tyrosine phophorylation of cellular proteins were detected in total cell lysates by immunoblot analysis (IB) with anti-phosphotyrosine (anti-pTyr) antibody (PY20). The blots were reprobed with anti-GFP, anti-BRK and anti-β- Tubulin antibodies as a loading control. (B) Tyrosine phosphorylated endogenous Dok1 as confirmed by anti-Dok1 immunoprecipitation (IP) followed by immunoblot analysis with anti-phosphotyrosine antibody and anti-Dok1(top panel). Immunoblot analysis of total cell lysates is showing the expression of Dok1, kinase activity of BRK-WT and BRK-YF, and β-tubulin as a loading control (bottom panel). (C) An *in vitro* kinase assay was performed using the active kinase, GST-BRK, and the substrate, GST-C-terminus Dok1, in the presence (+) or absence (−) of ATP. Tyrosine phosphorylation was detected using anti-phosphotyrosine antibody. The blots were reprobed with anti-BRK and anti-Dok1 antibody(bottom panel).

We transiently transfected the human embryo kidney (HEK) 293 cells with GFP-Dok1 in the presence or absence of constitutively active myc-tagged BRK (BRK-Y447F or BRK-YF). As a positive control, we used GFP-Sam68, a characterized substrate of BRK [29]. By immunoblotting with an anti-phosphotyrosine antibody PY20, we show that BRK-YF triggered strong tyrosine phosphorylation of GFP-Dok1, (Figure 1A, lane 5); likewise, GFP-Sam68, which migrates at a slower rate than GFP-Dok1, was also phosphorylated as expected (lane 6). The expression levels of GFP-Dok1 and GFP-Sam68 as well as those of myc-BRK-YF are shown in the bottom panels. These data show that overexpression of constitutively active BRK induces the phosphorylation of ectopically expressed GFP-Dok1.

We then examined whether ectopically expressed BRK could phosphorylate endogenous Dok1. To this end we transiently transfected either the kinase-dead BRK-K219M, BRK wild type (BRK-WT) or the constitutively active BRK-YF into HEK293 cells followed by immunoprecipitation and immunoblotting (Figure 1B). Using a phosphotyrosine antibody, we confirmed the phosphorylation of endogenous Dok1 in Dok1 immunoprecipitates from BRK-WT and BRK-YF cell lysates (Figure 1B, top panel, lanes 3 and 4). Strikingly, we observed a marked decrease in the levels of phosphorylation of the Dok1 protein in the immunoprecipitates from the BRKY447F-transfected cell lysates. No phosphorylation of Dok1 was detected in control cell lysates or lysates from BRK-KM-transfected cells (lanes 1 and 2, bottom), suggesting that BRK may directly phosphorylate Dok1 *in vivo*. The expression levels of Dok1 and activity of the transfected BRK variants (BRK-WT and BRK-YF) in the total cell lysates revealed strong phosphotyrosine staining as compared to either BRK-KM samples or the control lysates, as expected (Figure 1B, bottom). In light of these findings, we evaluated whether Dok1 was a direct substrate of BRK. In an *in vitro* kinase assay that was performed using glutathione S-transferase (GST)-tagged full-length BRK and the C-terminal region of Dok1 (GST-Dok1-CT)-terminal, we observed phosphorylation of GST-Dok1-CT in the presence of GST-BRK, indicating that Dok1 is a direct substrate of BRK (Figure 1C, lane 3, top panel). The activity of GST-BRK is shown by the presence of autophosphorylation (lanes 2 and 3). Together, these findings validate Dok1 as a bona fide BRK substrate.

### BRK phosphorylates Dok1 at tyrosine 362

**Figure 2:**
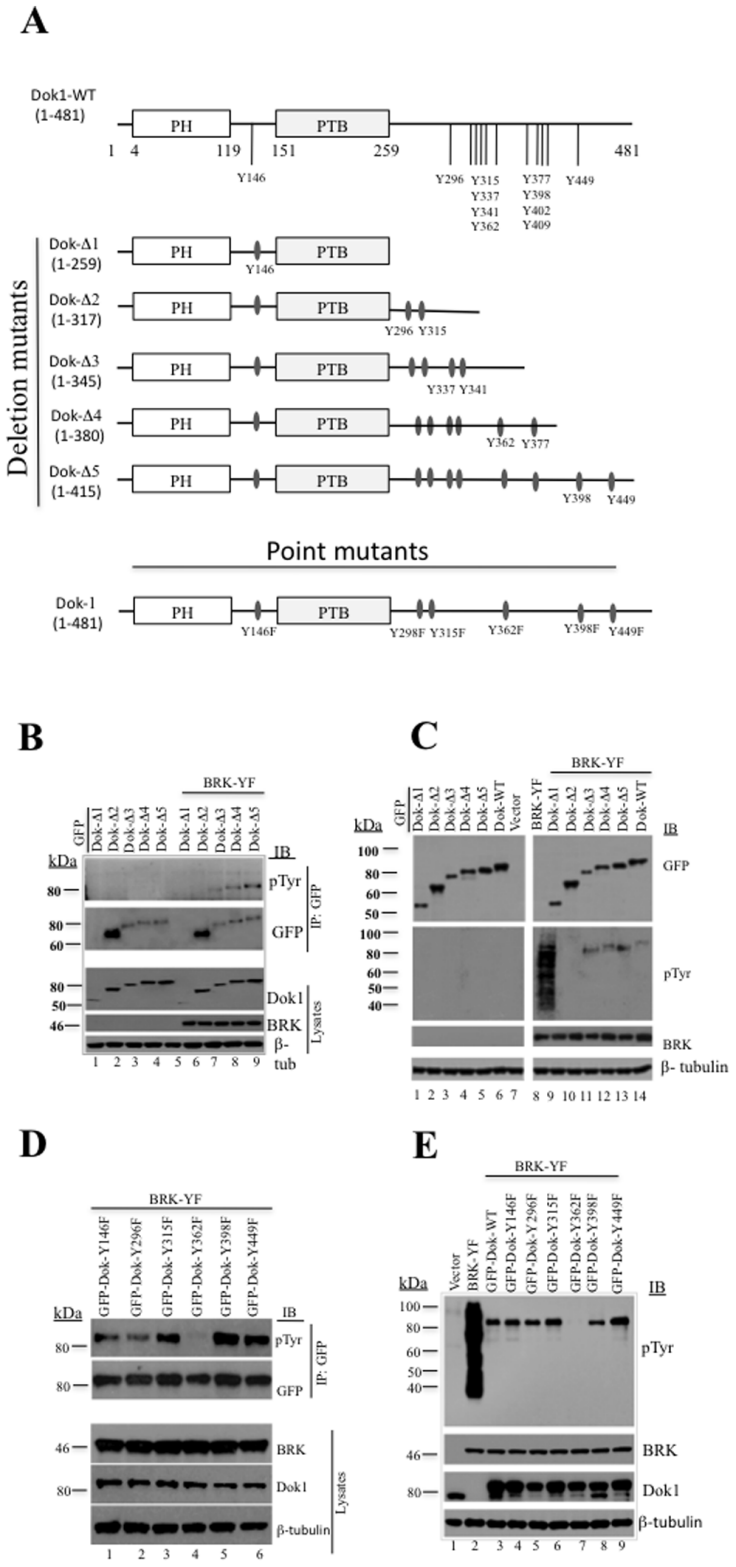
Constitutively active BRK phosphorylates Dok1 at Y362. (A) Schematic diagram of Dok1 showing different deletion and point mutants. (B) The Dok1 deletion mutants and BRK-YF were co-transfected in to HEK 293 cells, the cell were then subjected to immunoprecipitation with anti-GFP antibody followed by immunobloting analysis using anti-phosphotyrosine and anti-GFP antibodies (top panel). Lower panel shows the expression of different GFP-Dok1 deletion mutants, BRK (as input) and β-tubulin as a loading control. (C) Dok1 deletion mutants were transfected either alone or with BRK-YF into HEK 293 cells, the cell lysates were then subjected to immunobloting analysis using antibodies against Dok1, phosphotyrosine, BRK and β-tubulin as loading control. (D) HEK 293 cells were co-transfected with Dok1 point mutants and BRK-YF followed by immunoprecipitation with anti-Dok1 antibody and immunoblotting analysis using anti-phosphotyrosines and anti-Dok1 antibodies. Lower panel shows the expression of BRK, GFP-Dok1 mutants (as input) and β-tubulin as a loading control. (E) HEK 293 cells were cotransfected with BRK-YF and Dok1 point mutants or transfected with BRK-YF alone. Total cell lysates were analyzed by immunoblotting analysis with antibodies against phosphotyrosines, BRK, Dok1 and β-tubulin as loading control.

Dok1 is structurally composed of an N-terminal Pleckstrin Homology (PH) domain and an Insulin Receptor Substrate (IRS) Type PTB domain with a C-terminal segment rich in proline residues and also festooned with several tyrosine residues [47] (Figure 2A). To map the tyrosine(s) on Dok1 phosphorylated by BRK, we first generated five GFP-tagged deletion mutants of Dok1 (Dok1-Δ1 to Δ5) (Figure 2A). We transfected the Dok1 deletion mutants in the presence or absence of BRK-YF into HEK293 cells and then immunoprecipitated the Dok1 variants using anti-GFP antibodies. The immunoprecipitates were then analyzed by immunoblotting using anti-phosphotyrosine antibodies (PY20) (Figure 2B). We observed that the presence of BRK-YF induced the phosphorylation of all GFP-Dok1 variants, except for the Dok-Δ1 and Dok-Δ2 fragments, which harbor respectively Y146 or Y146 together with Y296 and Y315 (Figure 2B). Analysis of total cell lysates also corroborated the phosphorylation of the Dok1 mutants (Figure 2C). These data confirmed that BRK targets the tyrosine residues in the C-terminal of Dok1.

To determine which of the specific tyrosine residues along the C-terminal tail of Dok1 are targeted by BRK, we generated a series of 6 GFP-Dok1 mutants in which one of the following tyrosine residues, Y146, Y296, Y315, Y362, Y398 or Y449 was replaced by a phenylalanine (Figure 2A). Each construct was transiently transfected into HEK 293 cells that stably expressed the BRK-YF protein. The Dok1 mutants were then immunoprecipitated from the cell lysates with anti-GFP antibodies and analyzed by immunoblotting using PY20. As shown in Figure 2D, immunoblotting with the anti-phosphotyrosine antibodies revealed a robust phosphorylation of Dok1 wild type and all its mutants, except for GFP-Dok1 Y362F. The expression levels of phosphotyrosines, GFP Dok1 mutants and BRK-YF in the total cell lysates are shown in Figure 2E. As a whole, although transient co-transfection experiments showed a weak phosphorylation of GFP-Dok1 Y362F, our data support the notion that BRK induces the phosphorylation of Dok1 predominantly through tyrosine 362.

### BRK interacts with Dok1 via SH3 and SH2 binding

**Figure 3:**
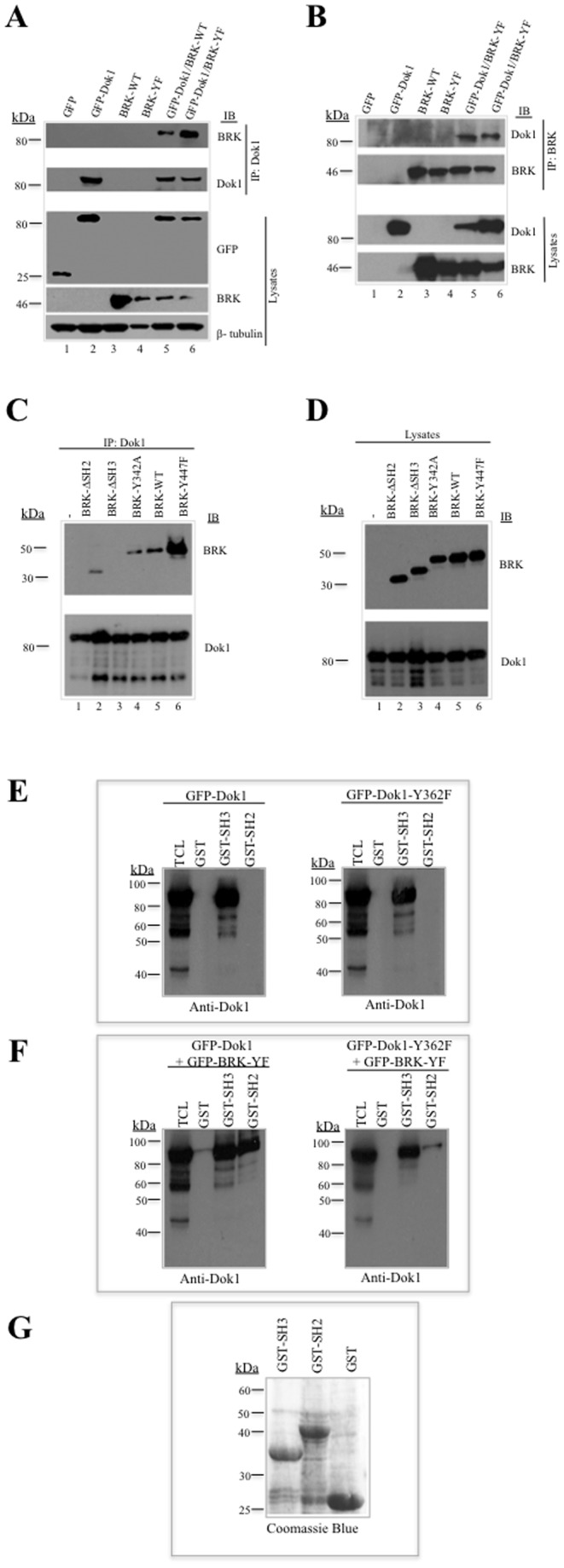
BRK interacts with Dok1 through the SH3 domain *in vivo* and *in vitro*. (A) HEK 293 cells were transfected with empty vector, Myc-BRK-WT, Myc-BRK-YF, GFP-Dok1 or co-transfected with Myc-BRK-WT/GFP-Dok1 or Myc-BRK-YF/GFP-Dok1 and subjected to immunoprecipitation with anti-Dok1 and immunoblotted with BRK and Dok1 (top 2 panels). The expression of cellular proteins was determined in total cell lysates by immunoblotting for GFP, BRK and β-tubulin as loading control. (B) BRK was immunoprecipitated with anti-BRK and subjected to immunoblotting analysis with anti-phosphotyrosine, anti-Dok1 and anti-BRK antibodies (top panels). Total cell lysates indicate the expression of BRK and Dok1 proteins. (C &D) HEK 293 cells were transfected with GFP-Dok1 alone or cotransfected with the idicated mutants of BRK and subjected to immunoprecipitation with anti-Dok1 followed by immunoblotting analysis with anti-BRK and anti-Dok1 antibodies. The cellular proteins were determined from the total cell lysates by immunoblotting analysis with anti-BRK and anti-Dok1 antibodies. (E) Overexpressed GFP-Dok1 or GFP-Dok1-Y362F in HEK 293 cell lysates from GFP-Dok1 or GFP-Dok1-Y362F expressing cells were subjected to pull-down assays with GST alone or recombinant GST-SH3 or GST-SH2 domain of BRK and immunoblotting analysis was performed with anti-Dok1 antibody. (F) GFP-Dok1/BRK-YF or GFP-Dok1-Y362F/BRK-YF cotransfected cohorts of HEK 293 cell lysates were subjected to pull-down assays with GST alone or GST-SH3 or GST-SH2 domain of BRK followed by immunoblotting with anti-Dok1 antibody. (G) Bacterially expressed GST, GST-SH3 and GST-SH2 domain of BRK proteins were detected via Coomassie blue staining.

BRK possesses three functional domains: SH3, SH2, and a catalytic domain. The SH3 domain binds to proline-rich regions typically with the PXXP motif, while the SH2 domain tends to bind to phosphorylated tyrosine residues. Previous studies have shown that the SH3 domain of BRK plays a pivotal role in substrate recognition and that the SH2 domain interacts with phosphorylated residues of BRK substrates. The C-terminus of Dok1 contains several proline residues and the entire polypeptide contains eight PXXP motifs. We therefore examined whether Dok1 interacts with BRK and whether this interaction is SH3- and/or SH2-dependent and direct. First we transfected GFP-Dok1 in the presence or absence of either BRK-WT or BRK-YF in HEK 293 cells and subjected the cell lysates to immunoprecipitation with antibodies against Dok1 and BRK. We found that BRK associated with Dok1 and the strongest association was observed in GFP-Dok1/BRK-YF samples (Figure 3A, lane 6). We also observed a reciprocal association of GFP-Dok1 in anti-BRK immunoprecipitates (Figure 3B). Our data suggest that both BRK and GFP-Dok1 interact.

Next, to map the binding domain of BRK, we co-expressed the BRK-WT, constitutively active BRK-YF and kinase-inactive BRK-Y342A, as well as BRK mutants lacking an SH2 domain (ΔSH2-BRK) or an SH3 domain (ΔSH3-BRK) with GFP-Dok1 in 293 cells. We found that GFP-Dok1 co-precipitated with BRK-WT, ΔSH2-BRK, BRK-Y342A and BRK-Y447F, but not with ΔSH3-BRK (Figure 3C). Analyses of the total cell lysates are shown in Figure 3D. Together, these results suggest that recognition of GFP-Dok1 was mediated primarily by SH3 domain interactions. Interestingly, BRK-Y447F displayed a marked increase in GFP-Dok1 binding (Figure 3A, B and C, lane 6).

To further confirm that the binding of Dok1 to BRK was governed by the SH3 domain and to demonstrate whether the SH2 domain preferentially binds to tyrosine phosphorylated Dok1, we performed glutathione S-transferase (GST) pulldown assays on cell lysates from HEK 293 cells transfected with GFP-Dok1 WT or GFP-Dok1Y362F alone or cotransfected with BRKY447F. Probing with an antibody against Dok1 revealed that in the absence of BRK Y447F, GFP-Dok1 WT and GFP-Dok1Y362F were able to interact with GST-BRK-SH3, but not with the GST-BRK-SH2 (Figure 3E). In the presence of BRK Y447F, in addition to SH3-binding, we also observed a strong interaction between GFP-Dok1 WT and BRK-SH2 domain (Figure 3F). However, the interaction between GFP-Dok1Y362F and BRK SH2 domain was markedly weaker than that of GFP-Dok1 WT (Figure 3F, right panel). This was predictable since we had showed in Figure 2D that BRK preferentially phosphorylates Dok1 on Y362. These data validate that BRK interacts with Dok1 through SH3 interactions and also suggest that the SH2 domain of BRK interacts predominantly with tyrosine-phosphorylated Dok1. Taken together, our data demonstrated the interaction between Dok1 and BRK under *in vivo* and *in vitro* conditions occurs via SH3 and also via SH2 binding on phosphorylated Y362 of Dok1.

### Inverse correlation between the levels of BRK and Dok1 in breast cancer cells

**Figure 4:**
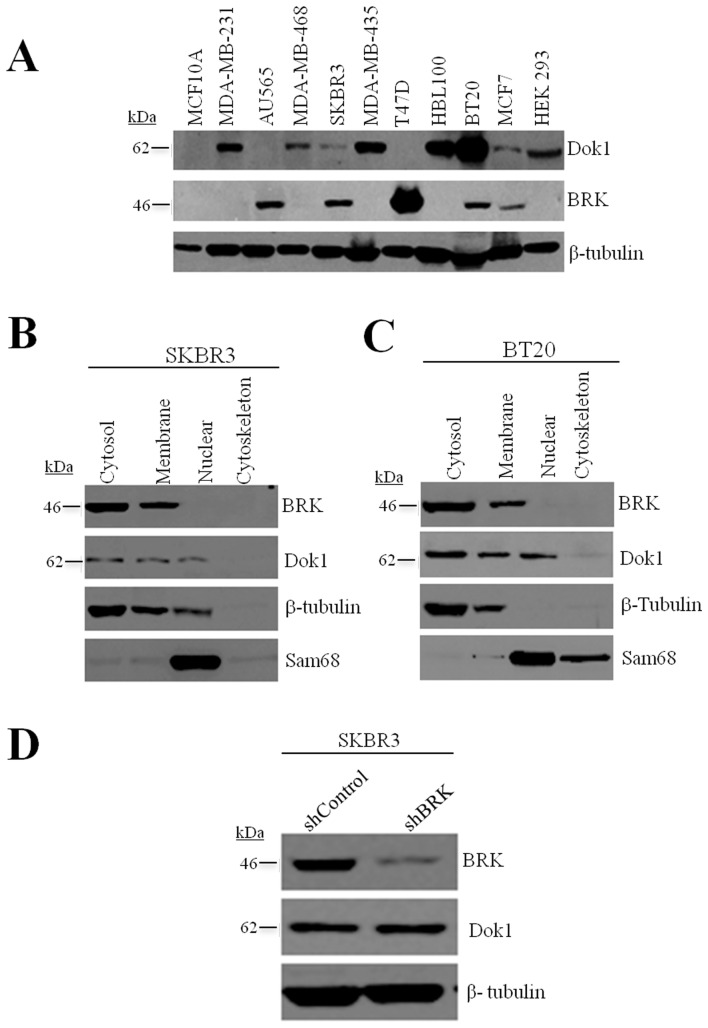
BRK and Dok1 are differentially overexpressed in the human breast cancer cell lines. (A) Cellular proteins were detected in total cell lysates by immunoblotting analysis with anti-Dok1 and anti-BRK antibodies. β-tubulin expression served as a loading control. (B & C) SKBR3 and BT20 cells were fractionated into the cytosolic, membrane, nuclear and cytoskeleton fractions and subjected to immunoblotting analysis for the detection of BRK and Dok1. β-tubulin and Sam68 were used as controls for the cytosolic/membrane and nuclear compartments, respectively. (D) Stable BRK knockdown was performed on parental breast cancer cell lines SKBR3 using shRNA lentiviral vector plasmids against BRK and analyzed as indicated.

Since Dok1 has been described as a candidate tumor suppressor [45], [48] and work from our laboratory and others indicate that BRK has oncogenic properties [28], [49], we opted to investigate the functional link between BRK and Dok1. We began by evaluating the expression of Dok1 in breast cancer cells in order to determine if there was any correlation between the expression profiles of both proteins. Using immunoblotting analysis, we examined the expression of Dok1 and BRK in nine breast cancer cell lines and in an immortalized mammary epithelial cell line, MCF-10A, as well as in the Human Embryonic Kidney 293 (HEK 293) cells. All the cell lines expressed detectable levels of Dok1, except for MCF-10A, AU565 and T47D. The strongest expression was observed in BT20 cells while weaker expression levels occurred in SKBRK3 and MCF7 (Figure 4A). BRK on the other hand was readily detectable in the breast cancer cell lines AU565, SKBR3, T47D, MCF7 and BT20, but not detectable in MCF-10A, MDA-MB-231, MDA-MB-435, MDA-MB-468 and HBL-100 (Figure 4A). The localization of BRK is predominantly cytoplasmic [29] and previous reports have shown that endogenous Dok1 was localized predominantly in the cytoplasm and plasma membrane [44], [50]. Using sub-cellular fractionation studies, on Dok1 and BRK-positive breast cancer cell lines, SKBR3 and BT20, we found that both BRK and Dok1 fractionated to the cytosolic and membrane fractions (Figure 4B and 4C). Since BRK and Dok1 were collected in the same cellular compartments and the expression levels of both proteins are inversely correlated, we investigated whether suppression of BRK expression by RNA interference could modulate the expression levels of Dok1 protein. As shown in Figure 4D, using short hairpin RNA (shRNA) against BRK in SKBR3 cells, we achieved a 60–70% knockdown of BRK; although, the suppression of BRK did not have any significant effect on Dok1 re-expression.

We previously showed that BRK is activated following stimulation with EGF [29]. We therefore investigated the effect of EGF stimulation on DOK1 expression in the presence or absence of BRK (Figure S2 in File S1). We stimulated SKBR3 breast cancer cell lines with EGF and observed peak activation of EGFR signalling at 5 minutes. We repeated the stimulation in BRK-positive as well as BRK negative (knockdown) SKBR3 cells and observed that while treatment with EGF suppressed endogenous Dok1 expression, a detectable increase in Dok1 levels was observed in BRK-knockdown cells (Figure S2 in File S1). Our data together indicate an inverse correlation between the expression of Dok1 and BRK that is partly regulated through EGF stimulation.

### Activated BRK downregulates Dok1 protein expression

**Figure 5:**
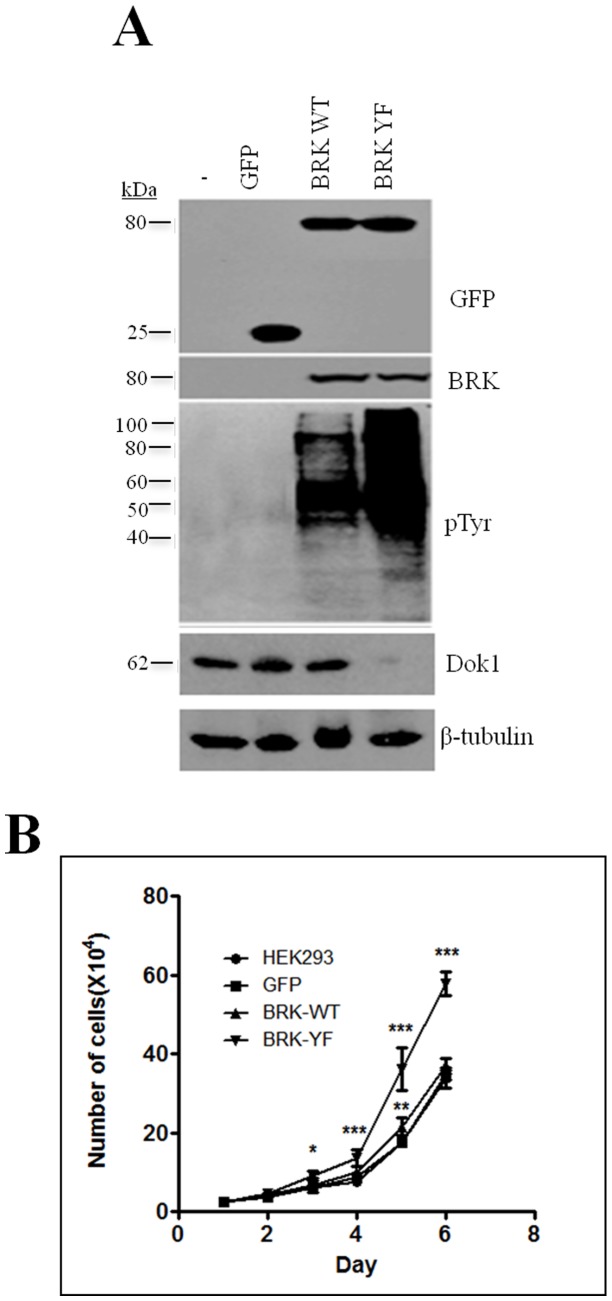
Constitutively active BRK downregulates Dok1 protein expression. (A) Immunobloting analysis of total cell lysates from HEK-293 stable cell lines is showing the expression of GFP alone' GFP-BRK-WT and GFP-BRK-YF (top panel), BRK (middle panel) and phosphorylated tyrosines (bottom panel). β-tubulin served as a loading control. (B) Immunobloting analysis of endogenous Dok1 in the stable HEK293 sublines. Expression of Dok1 was examined by immunoblotting analysis. (C) Characterization of cell proliferation in response to BRK-WT and BRK-YF. The *P*-values were determined for control and stably transfected cells and set at ***P≥0.0001, **P≥0.001 and *P≥0.05 for statistical significance.

The inverse correlation between BRK and Dok1 prompted us to further investigate whether BRK activation and over-expression could modulate the expression of Dok1 protein. In addition, previous studies have shown that oncogenic tyrosine kinases such as p210bcr-abl and v-Src downregulate Dok-1 in a kinase activity-dependent manner [44]. Since we recently reported that constitutively active BRK (BRK-Y447F) promotes tumor formation [28], we examined whether BRK-Y447F, like oncogenic Src, could downregulate endogenous Dok1. We used HEK 293 cells as a model to study the interaction between BRK and Dok1, since HEK 293 cells express high levels of Dok1, but express no endogenous BRK (Figure 4A). We generated three HEK 293 cell lines stably expressing GFP (empty control vector), GFP-BRK WT or GFP-BRK-YF by retroviral transduction. All stable cell lines expressed the transgene as determined by immunoblotting with the anti-GFP antibody (Figure 5A). Immunoblotting with anti-BRK confirmed the expression of GFP-BRK WT and Y447F and also validated the absence of BRK in HEK 293 cells. The BRK-transduced cells displayed elevated levels of phosphorylation of cellular targets, as visualized with an anti-phosphotyrosine antibody, PY20. Furthermore, as expected BRK-Y447F-transduced cells displayed activities that were significantly higher than those of BRK-WT (Figure 5A). We therefore evaluated the expression of Dok1 in all transduced cell lines and the parental control cell line and observed a significant reduction in the levels of Dok1 protein in the cells transduced with constitutively active BRK-Y447F compared to those in the BRK-WT, GFP alone, and in the parental cells (Figure 5A, bottom panels). Since Dok1 is a tumor suppressor and we observed a dramatic difference between the effects of BRK-WT and BRK-Y447F on Dok1 expression, we evaluated the growth rates of the stable cell lines. We found that the BRK-Y447F-transduced cells displayed significantly higher growth rates than the cells transduced with either BRK-WT or GFP alone (Figure 5B). Taken together, our data indicate that the catalytic activation of BRK is critical in its ability to downregulate endogenous Dok1 and that the observed suppression of Dok1 may contribute to BRK-promoted cell growth.

### Constitutively activated BRK diminishes the stability of Dok1 protein

**Figure 6:**
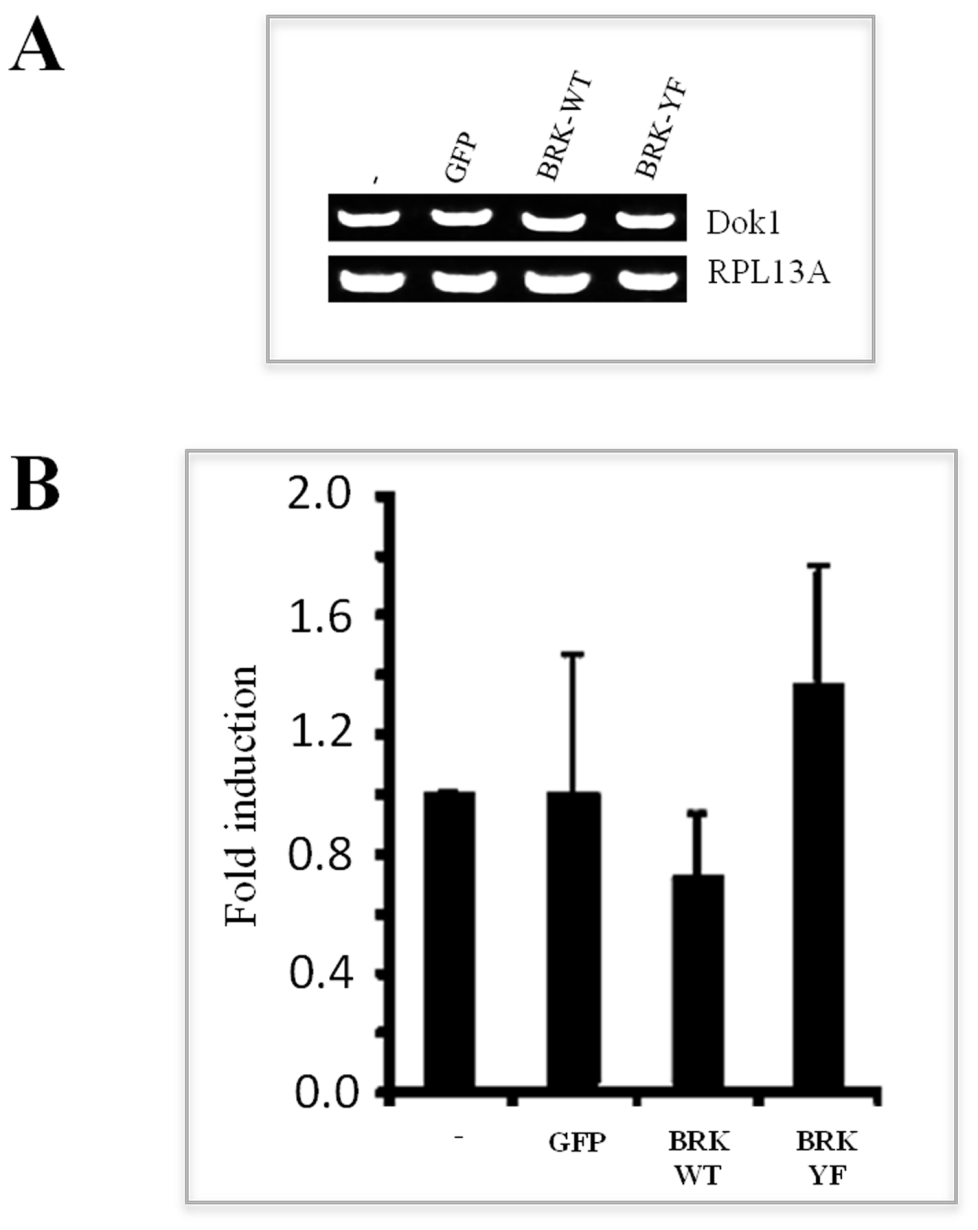
Constitutively active BRK does not affect the levels of Dok1 mRNA. (A & B) Total RNA was isolated from HEK 293 cells stably transduced with empty vector, GFP, GFP-BRK-WT and GFP-BRK-YF. Levels of Dok1 mRNA were then analyzed using RT-PCR (A) and qPCR (B). RPL13A gene was used as internal control. Error means are ± SEM of three biological repeats each having three technical repeats.

Since BRK induces the downregulation of Dok1, we then investigated the mechanisms of action of BRK in the downregulation of Dok1. Dok1 is a tumor suppressor and there are several potential mechanisms that account for the inactivation of tumor suppressors in cancer including those pertaining to the regulation of gene expression and post-translational events targeting protein stability [51], [52], [53], [54]. To identify the underlying mechanisms by which BRK-Y447F downregulates Dok1, we first evaluated the expression of Dok1 transcripts in BRK-transduced HEK 293 cells by both semi-quantitative RT-PCR and quantitative real-time qRT-PCR using Dok1-specific primers. As shown in Figure 6A and 6B, RT-PCR and qRT-PCR analyses revealed no significant difference between the levels of Dok1 mRNA levels in BRK-Y447F-transduced cells compared with cells stably expressing BRK-WT or the control cell lines. These results clearly indicate that BRK does not influence Dok1 mRNA levels, implying that BRK downregulates Dok-1 posttranscriptionally.

**Figure 7:**
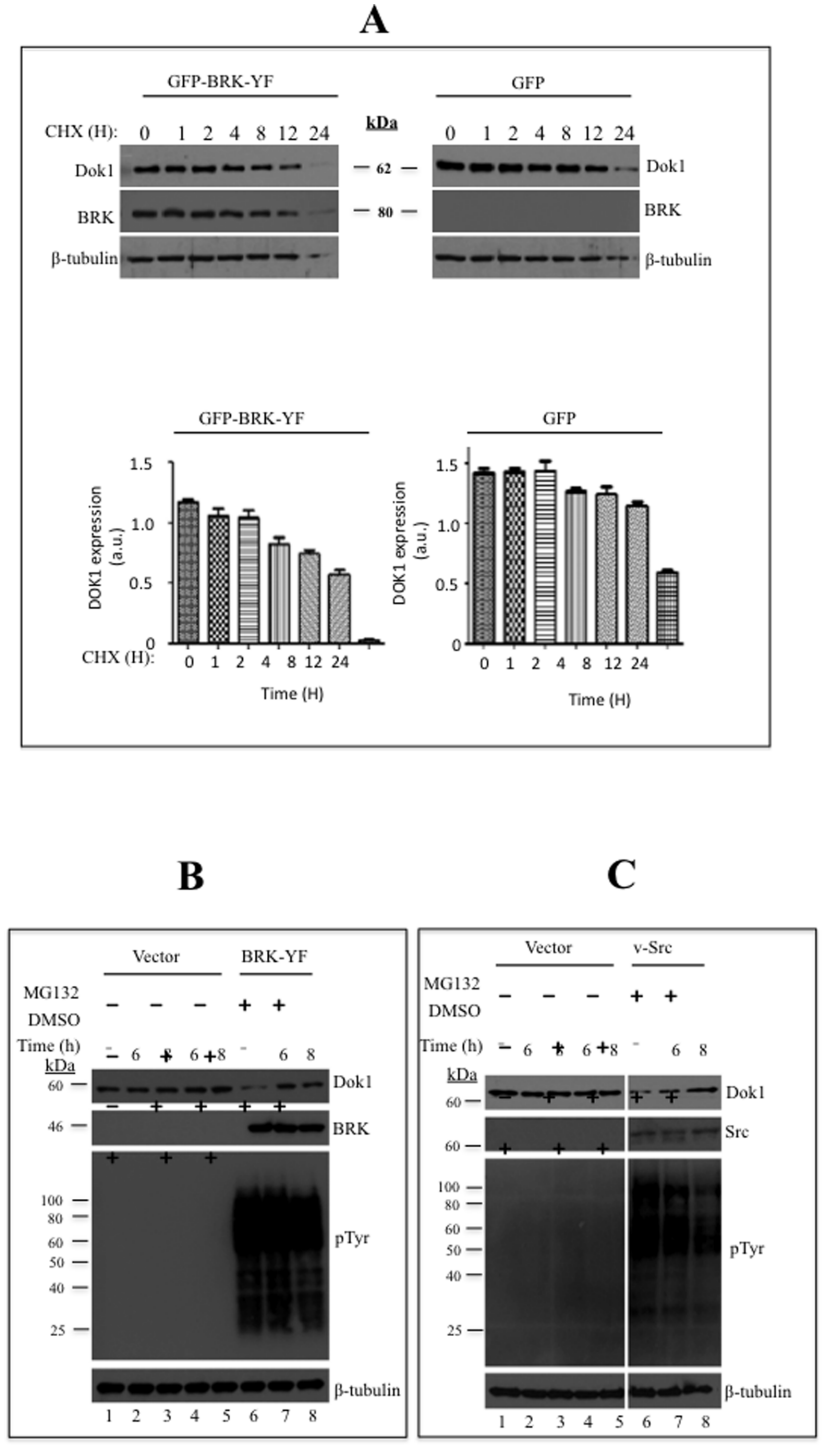
Activated BRK downregulates Dok1 by reducing its stability. (A) HEK 293 cells or HEK 293-BRK-YF stable cell line were treated with a protein synthesis inhibitor cyclohexamide (CHX: 200 µg/ml) for the indicated time points and then the cells were lysed and analyzed by immunoblotting for Dok1, BRK and β-tubulin as a loading control. (B) HEK 293 cells were stably transduced with HEK293-BRK-YF and treated with either a proteosome inhibitor MG132 (10 µM) or the vehicle DMSO as the control, at different time points (above the plot). Cellular proteins were determined in total cell lysates by immunoblotting analysis with anti-Dok1, anti-BRK, anti-phosphotyrosine antibodies. β-tubulin was used as a loading control. (C) Empty vector or V-Src was transiently transfected into HEK293 cells and the cells treated with a proteosome inhibitor MG132 (10 µM) and vehicle control DMSO for the indicated time points. Immunoblotting analysis of total cell lysates was performed to detect Dok1, v-Src, phosphotyrosines and β-tubulin served as a loading control. (D & E) HEK 293 cells were transfected with empty control vector or BRK-YF or v-Src and treated with MG132 (10 µM) and Lactacystin (5 µM) or control vehicle for 8 hours. Then the cell lysates were subjected to immunoblot analysis with anti-Dok1 antibody. β-tubulin as a loading control. (F) HEK293-BRK-YF stable cells were transiently cotransfected with Dok1 and HA-Ubiquitin plasmids and after 12 hours the cells were treated MG132 (10 µM) for an additional 8 hours. The total cell lysates were subjected to immunoprecipitation with anti-Dok1 followed by immunoblotting analysis with anti-HA and anti-Dok1 antibodies. The inputs were analysed as indicated.

Our results in Figure 6 led us to the hypothesis that BRK downregulates Dok1 by reducing the stability of Dok1 protein. To investigate this possibility, we examined the relative half-life of endogenous Dok1 protein in the presence or absence of BRK-Y447F. To this end, we treated various BRK-Y447F-transduced HEK 293 cells and control cells at various time points between 1 and 24 hours with cycloheximide (CHX, an inhibitor of protein synthesis). The cells were harvested periodically as indicated in Figure 7A. Western blotting of BRK-Y447F cell lysates showed that Dok1 protein levels were reduced by more than 50%, 75% and 95% at 8, 12 and 24 hours, respectively following treatment with CHX (Figure 7A, left panel). However, in the control samples (Figure 7A, right panel) the initial Dok1 protein level was reduced to half after 12 hours of treatment with CHX and a residual level of about 25% after 24 hours (Figure 7A, right panel). Statistical quantification of the levels of expression of Dok1 is provided in Figure 7A, lower panels. Our data thus indicate that Dok1 exhibited a relatively shorter half-life in cells transduced with BRK-Y447F compared with the control cells. These data indicate that Dok1 is a relatively stable protein with a physiological half-life of about 12 hours and that the stability of the Dok1 protein is compromised in the presence of constitutively active BRK.

### BRK downregulates Dok1 via proteasomal degradation

We then explored the possibility that the BRK-induced downregulation of Dok1 is mediated by the ubiquitin proteasome pathway (UPP). UPP plays a well-characterized key role in protein stability by eliminating several intracellular proteins in eukaryotes via degradation [55]. To investigate whether Dok1 is regulated by BRK-Y447F via UPP associated degradation, we first examined Dok1 protein levels in the presence or absence of the peptide-aldehyde proteasome inhibitor MG132 (carbobenzoxyl-L-leucyl-L-leucyl-L-leucine). MG132 is a specific proteasome inhibitor that binds reversibly to the N-terminal Thr residue of the β1 subunit within the 26S proteasome [56]. We used the oncogenic v-Src as a positive control in these experiments since its effect on Dok1 stability in the presence of MG132 has been characterized [57]. First we examined the effect of MG132 on Dok1 stability in the control HEK 293 cells transduced with empty vector. The cells were treated with or without MG132 for 6 or 8 hrs. Similar treatment with dimethyl sulfoxide (DMSO) served as a negative control (Figure 7B). We observed that MG132 treatment led to an increase in Dok1 protein levels in HEK293 cells (Figure 7B, lanes 4 and 5). The increase in Dok1 levels as a result of MG132 treatment indicates that the UPP associated degradation is indeed involved in regulating Dok1 protein turnover. We then transfected HEK 293 cells with plasmids expressing either BRK-Y447F or v-Src, and then treated the cells with MG132 or DMSO at the indicated intervals (Figure 7B, C). We observed a strong increase in the levels of the Dok1 protein following MG132 treatment in cells transfected with either BRK-Y447F or v-Src (Figure 7B, C). The presence of MG132 did not affect the activity of BRK-Y447F as indicated by comparable anti-phosphotyrosine staining (Figure 7B, lanes 6, 7 and 8). Surprising, the levels of tyrosine phosphorylation in v-Src-transfected cells were slightly lowered in the presence of MG132 (Figure 7C, lanes 6, 7 and 8). These findings suggest that the increase in Dok1 protein levels was the result of an inhibition of the proteasomal proteases by MG132. To substantiate these findings, we treated BRK-Y447F and v-Src-transfected cells for 8 hrs with lactacystin, a proteasome inhibitor that binds covalently to the 26S proteasome [58]. Immunoblot analysis revealed that treatment with lactacystin, akin to MG132, resulted in the stabilization of Dok1 protein in both BRK-Y447F and v-Src transfected cells (Figure 7D, E). These findings support our notion that both BRK and v-Src render Dok1 unstable and increasingly prone to degradation via the UPP. Finally, we examined whether the ubiquitination machinery directly mediates the BRK-induced downregulation of Dok1. Plasmids expressing GFP-Dok1, myc-BRK-Y447F and HA-ubiquitin were transfected into HEK 293 cells. The cells were then treated with either the protease inhibitor, MG132 or DMSO as a negative control. We performed an immunoprecipitation assay using anti-Dok1 antibodies followed by immunoblotting using anti-HA antibodies. Our data showed a significantly enhanced high molecular weight smear of molecules conjugated to the mCherry-Dok1 immunoprecipitates in the presence of MG132 (Figure 7F, lanes 6 and 7) compared with the controls in BRKY44F-expressing cells (lanes 1–5). In the presence of BRK-Y447F, ectopic of GFP-Dok1 and HA-Ub, in the presence or absence of MG132, resulted in no ubiquitination of Dok1 (Figure S3). It is worth noting that treatment with MG132 in the presence of GFP-BRK-Y447F had a greater impact on endogenous Dok1 levels (Figure 7D) than the ectopically expressed GFP-Dok1 levels (Figure 7F); however, the reason for these discrepancies is not obvious to us. Our findings as a whole strongly support the notion that BRK destabilizes Dok1 by promoting its ubiquitination, and an eventual degradation via the ubiquitin-proteasome pathway.

### Overexpression of Dok1 suppresses BRK-induced cell proliferation and migration

**Figure 8:**
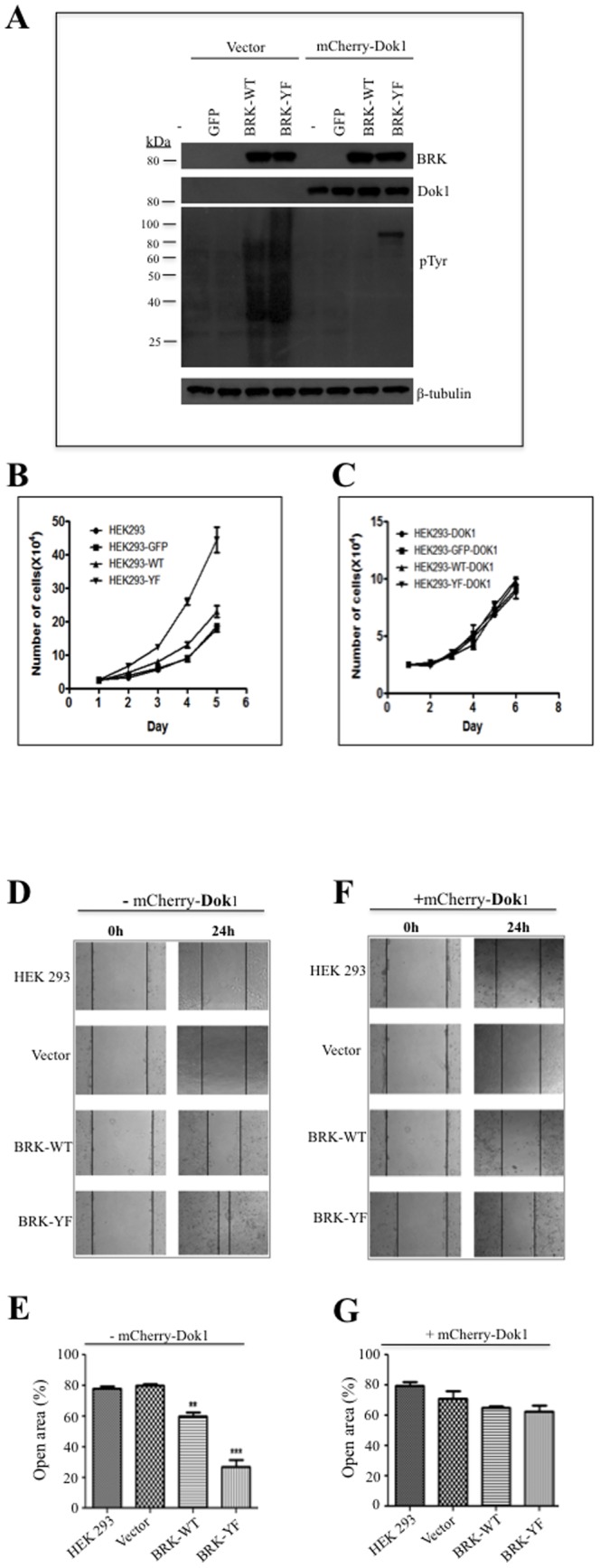
Dok1 inhibits BRK-induced cell proliferation and migration. (A) HEK 293 stable sub-cell lines were transduced with mCherry-Dok1 using adenoviral vector. Cellular proteins were detected in total cell lysates by immunoblotting analysis with anti-BRK, anti-Dok1, and anti-phosphotyrosine antibodies. β-tubulin served as a loading control. (B & C) HEK 293 stable cells were transduced with or without mCherry-Dok1adeno-vector and were monitored for cell proliferation. (D & E) Cell migration determined by the healing of a fixed wound area induced in the different HEK 293 stable transfectant cells. The percentage of open area at 24 h is plotted. (F & G) Cell migration analysis was performed with the indicated stable cell lines expressing mCherry-Dok1 or an empty vector. The assay was based on the rate of wound closure in the scratched cells. The percentage of open area at 24 hours is plotted. The migration assay was performed in three independent experiments. Data are means ± standard errors. Statistics: and **P≥0.001 and ***P≥0.0001.

Dok1 is a tumor suppressor and several studies have concurred that the overexpression of Dok1 suppresses cell proliferation and migration [45], [48], [59], [60]. Ectopic expression of Dok1 has been shown to inhibit cell proliferation and transformation induced by oncogenic tyrosine kinases, including the p210^bcr-abl^ and Src family kinases [57], [60]. Previously, we along with others showed that BRK overexpression and activation enhanced cell proliferation, cell migration and tumor formation [26], [28], [33], [61], [62], [63]. To test whether Dok1 can also modulate the oncogenic properties of BRK, we evaluated the effect of Dok1 on BRK-induced cell proliferation and migration. HEK 293 cells stably expressing GFP alone, GFP-BRK-WT and GFP-BRKY447F were infected with adenoviruses expressing mCherry-Dok1 (Figure 8A). In the absence of mCherry-Dok1, cells stably expressing GFP-BRKY447F displayed a significantly higher growth rate compared with GFP-BRK-WT expressing cells as well as the control cell lines (Figure 8B). Remarkably, the introduction of Dok1 resulted in a dramatic decrease in the rate of growth of the BRK-Y447F-transduced cells, similar to the levels exhibited by the control and BRK-WT cells (Figure 8C). Similar results were obtained with BRK-negative cells lines MDA-MB-231 stably expressing BRK variants BRK-Y447F or BRK-WT (Figure S4 in File S1). These data indicate that the overexpression of Dok1 suppresses BRK-induced cell proliferation.

Finally, we employed wound healing assays to assess the effect of Dok1 on BRK-induced cell migration using the same set of cell lines described in Figure 8B and C. The cell surfaces were scratched and photo-micrographs taken at different time intervals between 0 and 24 hours. Figure 8D and F show representative images taken at 0 and 24 hours. The results showed that the overexpression of BRK-Y447F accelerated the wound healing process as observed by the reduced size of the wounded area after 24 hours compared to either the BRK-WT or control samples (Figure 8D). Furthermore, the ectopic expression of Dok1 reduced the migration rates of the BRK-Y447F cells to near control rates (Figure 8F). The results are quantified in Figure 8E and G. These results were reproduced using MDA-MB-231 cells stably expressing the BRK variants (Figure S5 in File S1). Taken together, these data confirm the anti-oncogenic properties of Dok1 and suggest that downregulation of Dok1 is one of the mechanisms by which BRK manifests its oncogenic function.

## Discussion

We recently showed that constitutive activation of BRK promotes cell proliferation and migration as well as tumor formation, validating the proto-oncogenic function of BRK [28].; however, the molecular mechanisms dictating the tumorigenic role of BRK are poorly understood. An increasing number of studies have reported an inhibitory function of oncogenic tyrosine kinases towards overcoming cellular and physiological constraints promoted by tumor suppressors [64], [65], [66], [67], [68]. Members of the Dok1 family have been characterized as negative regulators of cell transformation induced by oncogenic tyrosine kinases [45]. In the present study, we demonstrate for the first time that BRK mediates its oncogenic function at least in part by downregulating the tumor suppressor Dok1. We show that: 1) BRK interacts and phosphorylates Dok1 predominantly at tyrosine 362; 2) the levels of BRK and Dok1 in breast cancer cells are inversely correlated; 3) activated BRK promotes Dok1 protein downregulation via ubiquitin proteasome degradation; and 4) Dok1 is a negative regulator of BRK-induced cell proliferation and migration.

Dok1, also known as p62dok, is the prototypical member of a family of 7 adaptor proteins comprising Dok1 to Dok7. Since p62Dok was first identified as a substrate of p210bcr-abl, v-Abl [47], [69] and many other protein tyrosine kinases, it was therefore termed Dok, for downstream of tyrosine kinase [43], [44], [45], [70], [71]. Dok1 was functionally identified as a tumor suppressor based on several studies that demonstrated an antagonizing role of the adaptor protein towards p210bcr-abl-mediated cell transformation *in vivo* [59], [72]. An understanding of the physiological tumor suppressor role of Dok1 emerged from mice studies, revealing a significantly accelerated onset of the p210bcr-abl-induced chronic myelogenous leukemia (CML), a myeloproliferative disorder of the hematopoietic stem cell, upon Dok1 inactivation [59], [72]. In addition, mice with combined knockouts of Dok1, Dok2, and Dok3 developed aggressive histiocytic sarcoma [73] or lung adenocarcinoma [74].

The cytoplasmic protein Dok1 is functionally characterized by an N-terminal pleckstrin homology (PH) domain that allows anchorage to the membrane, followed by a phosphotyrosine-binding domain that is involved in protein-protein interactions, and a C-terminal region rich in tyrosine, proline and serine residues [45]. The function of Dok1 is regulated upon phosphorylation by a variety of receptor and non-receptor tyrosine kinases including the Src tyrosine kinase family members Lck and Fyn [75], as well as tyrosine kinases such as Tec and Bcr-Abl [43], [76], [77], [78], [79]. It has also been demonstrated that Src phosphorylates Dok1 and prevents its entry into the nucleus [44]. Recently, Takeda et al. identified Dok1 as a substrate of several tyrosine kinases including BRK [39]. In the present study we provide evidence that Dok1 interacts with and is a direct substrate of BRK. In cells stably expressing BRK, ectopically expressed Dok1 is phosphorylated preferentially on Y362. We noted that co-expression of BRK and Dok1 also resulted in the phosphorylation at other tyrosines, besides Y362, although the phosphorylation signal was weak. The reasons for these discrepancies in the phosphorylation pattern of Dok1 by BRK were not obvious. It is tempting to speculate that at steady state levels, BRK is more specific in its phosphorylation of Dok1. It was previously shown that Y361, and Y450 of murine Dok1, equivalent to Y362, and Y449 of its human homologue, are all direct phosphorylation site of Src [44], [80]. Phosphorylation at Y362 is not surprising since Y_361_DEP motif in murine Dok1 fits the optimal consensus phosphorylation site for Src [80]. However, the LY_450_QSV is not optimal for Src phosphorylation, although the pYXXV motif has been shown to be an optimal binding site for the c-Src tyrosine kinase SH2 domain [80]. Like Src, it is therefore possible that BRK could also phosphorylate unpredictable sites, based on the apparent heterogeneity of the consensus motif.

The *Dok1* gene localizes to human chromosome 2p13, a locus that is prone to genetic alterations in various human tumors [81], [82], [83]. Dok-1, Dok-2, and Dok-3 proteins are highly expressed in hematopoietic cells [72] [84]. In addition, higher expression levels of Dok1 were detected in serous epithelial ovarian cancer as compared to normal tissues and this overexpression significantly correlated with disease-free survival of serous epithelial ovarian cancer patients [85]. *Dok1* was also shown to be repressed in other forms of cancer including head and neck cancer (HNC), lung, liver, and gastric cancers, likewise in Burkitt's lymphoma [86], [87], [88]. Dok1 repression was suggested to been a consequence of aberrant promoter hypermethylation [86], [87], [88], although surprisingly no expression studies of Dok1 were found in the literature to support these findings. We evaluated the correlation between Dok1 and BRK in breast cancer cell lines, because of the over expression of BRK in majority of breast cancer [28], [49]. We observed an inverse correlation between the expression patterns of the two proteins, although the knockdown of BRK resulted in only a slightly elevated levels of Dok1 in SKBR3 cells. However, it is not yet known whether the expressions of Dok1 and BRK in breast cancers are regulated epigenetically via promoter hypermethylation; although, there is evidence of BRK promoter hypomethylation in cisplatin resistant ovarian cancer cells [89].

In the present study we also show that both BRK and Dok1 are predominantly expressed as cytoplasmic/membrane proteins (Figure 4B, C) as was previously reported [29], [49], [77], [79], [90]. A proportion of both proteins also localize in the nucleus [29], [44]. Others and we reported that BRK phosphorylates nuclear Sam68 and promotes its subcellular relocalization [29], [49]. Dok1 on the other hand was recently shown to shuttle between the nucleus and cytoplasm in a mechanism that is regulated by external stimuli and Src phosphorylation [44]. Further research is needed to determine whether BRK also regulates the subcellular localization of Dok1.

Both p210bcr-abl and v-Src have been shown to downregulate Dok-1 in a kinase activity-dependent manner [44]. BRK was recently shown to phosphorylate Cbl, inducing its auto-ubiquitinination and degradation through the ubiquitin-proteasome pathway [91]. In the present study, we found that the constitutively active BRK also induced the degradation of Dok1, but had no effect on its transcript (Figures 5, 6 and 7). Intriguingly, in comparison to BRK-YF, BRK WT induced little to no effect on Dok1 protein levels. This is reflective of a kinase dependent mechanism by which BRK downregulates Dok1 protein levels. This however raises the critical question as to how endogenous BRK is able to regulate Dok1 protein levels. We previously showed that the ability of BRK to phosphorylate its endogenous substrate Sam68 in breast cancer cells was significantly enhanced by stimulation with epidermal growth factor (EGF) [29]. Dok1 is a scaffolding protein and in the present study we have shown that BRK interacts with Dok1 via its SH3 domain (Figure 3E). It is conceivable that EGF stimulation may lead to BRK activation followed by its interaction, phosphorylation and therefore destabilization/degradation of Dok1 as shown in Figure S2 in File S1. It is worth noting is that the activation of protein tyrosine kinases via the transient treatment of fibroblast cells with platelet-derived growth factor (PDGF) did not induce a decrease in Dok1 expression [57]. But this effect of BRK on Dok1 may have been achieved through prolonged stimulation of BRK leading to its activation as demonstrated in the current work. It is not clear at this stage whether the effect of BRK was direct. We showed that tyrosine phosphorylated Dok1 interacts with the SH2 domain of BRK. How this interaction affects accessibility to the proteasomal machinery is not known. However, it is possible that activated BRK may have triggered other cellular events that culminated in other posttranslational modifications promoting Dok1 degradation. Usually, polyubiquitin chains are covalently bound to lysine residues in proteins targeted for degradation [55]. Though we conclude that the BRK-induced degradation of Dok1 is via the ubiquitin-proteasomal pathway, at this stage we did not provide direct evidence that this is a lysine-dependent mechanism. Ideally, a Dok1 substrate in which all lysine residues have been mutated to arginines will serve as a better negative control for the study of BRK-induced ubiquitination of Dok1. We did not test this possibility since studies with other oncogeneic tyrosine kinases have observed only a modest increase in the expression levels of the lysine-less mutant [57], raising the possibility that other potential posttranslational modifications might be involved in the ubiquitination process.

Finally, we provided a functional link between Dok1 inactivation and the regulation of BRK-induced cellular processes such as cell proliferation and migration. Dok1 itself has been shown to inhibit mitogenic signaling and cell proliferation, and to antagonize leukemogenesis, but paradoxically, it also promotes cell spreading, motility, and apoptosis [43], [59], [72], [92], [93]. Inhibition of Dok1 expression has been associated with enhanced cell proliferation [48], [94]. We found in Figure 8B and C that activated BRK-induced proliferation of stable HEK 293 cells was inhibited in the presence of overexpressed Dok1. Similarly, Dok1 suppressed BRK-induced migration of these cells. Taken together, our findings suggest that Dok1 is a negative regulator of the BRK-promoted oncogenic cellular processes, in particular cell migration and proliferation. Further studies are needed to comprehensively elucidate the physiological implications of Dok1 and other members of the DOK gene family in BRK-regulated mammary tumorigenesis using for instance xenograph mouse models.

In summary, our data show that Dok1 is phosphorylated and targeted for ubiquitin-proteasomal degradation, a process that is critical for the sustenance of BRK-induced cell migration and proliferation. Further in vivo studies are required to support a model in which Dok1 impacts BRK-driven tumorigenesis and metastasis.

## Supporting Information

File S1 **Supporting Figures. Figure S1. Constitutively active form of BRK mutant (BRK-YF) shows maximum kinase activity.** HEK 293 cells were transiently transfected with empty control vector (-) GFP-BRK-WT, GFP-BRK-KM or GFP-BRK-YF followed by immunoblotting analysis using anti-GFP and anti-phosphotyrosines antibodies. **Figure S2. The knock down of BRK in SKBR3 cells restores DOK1 protein level.** (A) SKBR3 cells were treated with EGF (100 ng/ml) for 0, 5, 10, 15 and 30 minutes and then subjected to immunoblot analysis for the detection of phosphotyrosines and β-tubulin (as a loading control). (B and C) SKBR3 and stable BRK knock down SKBR3 cells were treated with or without EGF (100 ng/ml) for 15 minutes. Total cellular proteins were determined from the cell lysates by performing immunoblot analysis with anti-BRK and anti-DOK1 antibodies. β-actin served as a loading controls and the DOK1 expression was quantified and shown in a bar diagram. **Figure S3. Dok1 is not ubiquitinated in the absence of BRK.** HEK293 cells were transiently co-transfected with GFP-Dok1, HA-ubiquitin and empty myc vector and incubated in the presence or absence of the proteasomal inhibitor, MG132 (10 µM) for 8 hours. Cell Lysates were subjected to immunoprecipitation with anti-Dok1 antibody and immunoblotting was performed with antibodies against HA and Dok1 (top panel). Total cell lysates were subjected to immunoblotting with antibodies against Dok1, BRK and β-tubulin as loading control. **Figure S4. Dok1 inhibits BRK-induced cell proliferation in MDA-MB 231 cells.** (A&B) MDA-MB 231 stable cells were transduced with or without mCherry-Dok1adeno-vector and were monitored for cell proliferation. **Figure S5. Dok1 inhibits BRK-induced cell migration in MDA-MB 231 cells.** (A & B) MDA-MB 231 stable cells were transduced with or without mCherry-Dok1adeno-vector and were monitored for cell migration based on the healing of the wound area. The percentage of open area at 24 hours is plotted. (C & D) Cell migration analysis was performed with the indicated stable cell lines expressing mCherry-Dok1 or an empty vector. The assay was based on the rate of wound closure in the scratched cells. The percentage of open area at 24 hours is plotted. The migration assay was performed in three independent experiments. Data are means ± standard errors. Statistics: *P≥0.05 and **P≥0.001.
(PPT)
