# Correction: BRK Targets Dok1 for Ubiquitin-Mediated Proteasomal Degradation to Promote Cell Proliferation and Migration

**DOI:** 10.1371/journal.pone.0098814

**Published:** 2014-05-22

**Authors:** The PLOS ONE Staff

Figure 7 is incorrect. The authors have provided a corrected version here. The publisher apologizes for this error.

**Figure 7:**
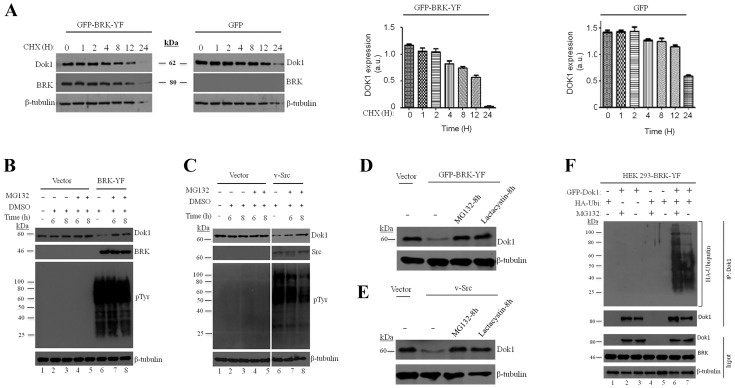
Activated BRK downregulates Dok1 by reducing its stability. (A) HEK 293 cells or HEK 293-BRK-YF stable cell line were treated with a protein synthesis inhibitor cyclohexamide (CHX: 200 µg/ml) for the indicated time points and then the cells were lysed and analyzed by immunoblotting for Dok1, BRK and β-tubulin as a loading control. (B) HEK 293 cells were stably transduced with HEK293-BRK-YF and treated with either a proteosome inhibitor MG132 (10 µM) or the vehicle DMSO as the control, at different time points (above the plot). Cellular proteins were determined in total cell lysates by immunoblotting analysis with anti-Dok1, anti-BRK, anti-phosphotyrosine antibodies. β-tubulin was used as a loading control. (C) Empty vector or V-Src was transiently transfected into HEK293 cells and the cells treated with a proteosome inhibitor MG132 (10 µM) and vehicle control DMSO for the indicated time points. Immunoblotting analysis of total cell lysates was performed to detect Dok1, v-Src, phosphotyrosines and β-tubulin served as a loading control. (D & E) HEK 293 cells were transfected with empty control vector or BRK-YF or v-Src and treated with MG132 (10 µM) and Lactacystin (5 µM) or control vehicle for 8 hours. Then the cell lysates were subjected to immunoblot analysis with anti-Dok1 antibody. β-tubulin as a loading control. (F) HEK293-BRK-YF stable cells were transiently cotransfected with Dok1 and HA-Ubiquitin plasmids and after 12 hours the cells were treated MG132 (10 µM) for an additional 8 hours. The total cell lysates were subjected to immunoprecipitation with anti-Dok1 followed by immunoblotting analysis with anti-HA and anti-Dok1 antibodies. The inputs were analysed as indicated.
