# Supplementary material for: BRK Targets Dok1 for Ubiquitin-Mediated Proteasomal Degradation to Promote Cell Proliferation and Migration
Source: PLoS One. 2014 Feb 11;9(2):e87684. doi: 10.1371/journal.pone.0087684 (PMC3921129; doi:10.1371/journal.pone.0087684)
Supplement: File S1 — Supporting Figures. Figure S1. Constitutively active form of BRK mutant (BRK-YF) shows maximum kinase activity. HEK 293 cells were transiently transfected with empty control vector (-) GFP-BRK-WT, GFP-BRK-KM or GFP-BRK-YF followed by immunoblotting analysis using anti-GFP and anti-phosphotyrosines antibodies. Figure S2. The knock down of BRK in SKBR3 cells restores DOK1 protein level. (A) SKBR3 cells were treated with EGF (100 ng/ml) for 0, 5, 10, 15 and 30 minutes and then subjected to immunoblot analysis for the detection of phosphotyrosines and β-tubulin (as a loading control). (B and C) SKBR3 and stable BRK knock down SKBR3 cells were treated with or without EGF (100 ng/ml) for 15 minutes. Total cellular proteins were determined from the cell lysates by performing immunoblot analysis with anti-BRK and anti-DOK1 antibodies. β-actin served as a loading controls and the DOK1 expression was quantified and shown in a bar diagram. Figure S3. Dok1 is not ubiquitinated in the absence of BRK. HEK293 cells were transiently co-transfected with GFP-Dok1, HA-ubiquitin and empty myc vector and incubated in the presence or absence of the proteasomal inhibitor, MG132 (10 µM) for 8 hours. Cell Lysates were subjected to immunoprecipitation with anti-Dok1 antibody and immunoblotting was performed with antibodies against HA and Dok1 (top panel). Total cell lysates were subjected to immunoblotting with antibodies against Dok1, BRK and β-tubulin as loading control. Figure S4. Dok1 inhibits BRK-induced cell proliferation in MDA-MB 231 cells. (A&B) MDA-MB 231 stable cells were transduced with or without mCherry-Dok1adeno-vector and were monitored for cell proliferation. Figure S5. Dok1 inhibits BRK-induced cell migration in MDA-MB 231 cells. (A & B) MDA-MB 231 stable cells were transduced with or without mCherry-Dok1adeno-vector and were monitored for cell migration based on the healing of the wound area. The percentage of open area at 24 hours is plotted. (C & D) Cell migration analys [file pone.0087684.s001.ppt]

## Slide 1
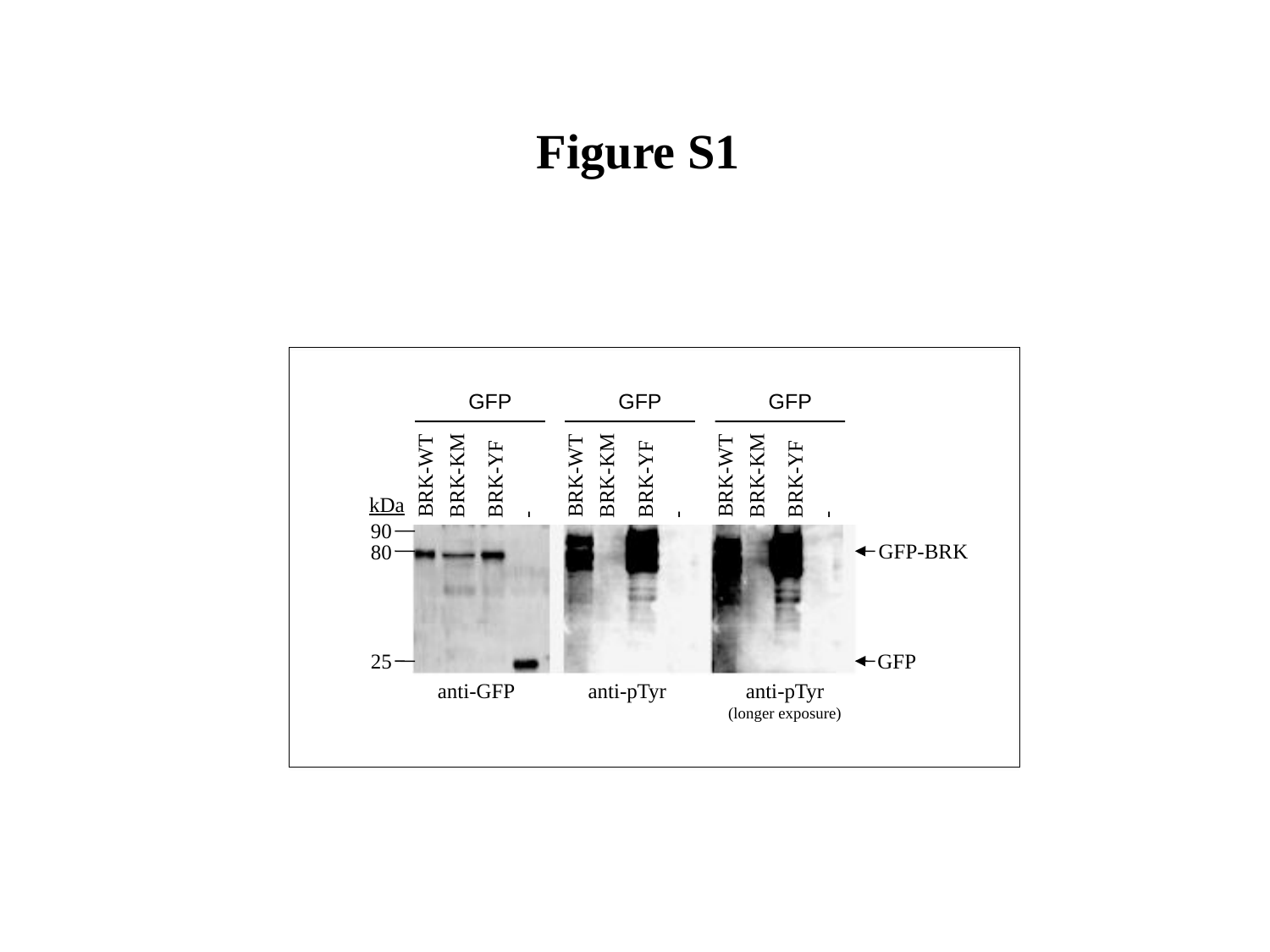

Figure S1
GFP
GFP
GFP
BRK-WT
BRK-KM
BRK-WT
BRK-KM
BRK-WT
BRK-KM
BRK-YF
BRK-YF
BRK-YF
kDa
-
-
-
90
GFP-BRK
80
25
GFP
anti-GFP
anti-pTyr
anti-pTyr
(longer exposure)

## Slide 2
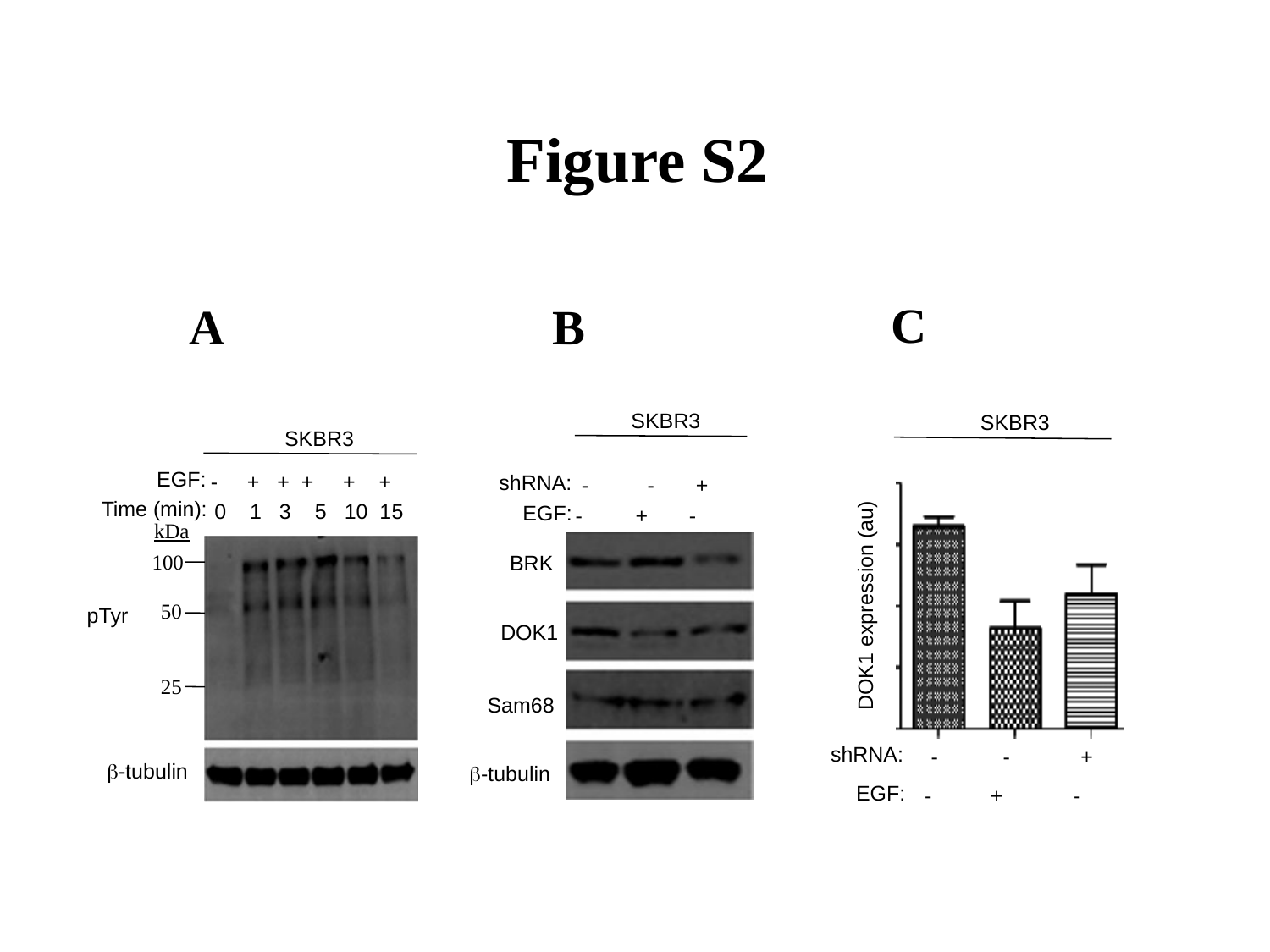

Figure S2
C
A
B
SKBR3
SKBR3
SKBR3
EGF:
- + + + + +
shRNA:
- - +
Time (min):
0 1 3 5 10 15
EGF:
- + -
kDa
100
BRK
DOK1 expression (au)
50
pTyr
DOK1
25
Sam68
shRNA:
- - +
β-tubulin
β-tubulin
EGF:
- + -

## Slide 3
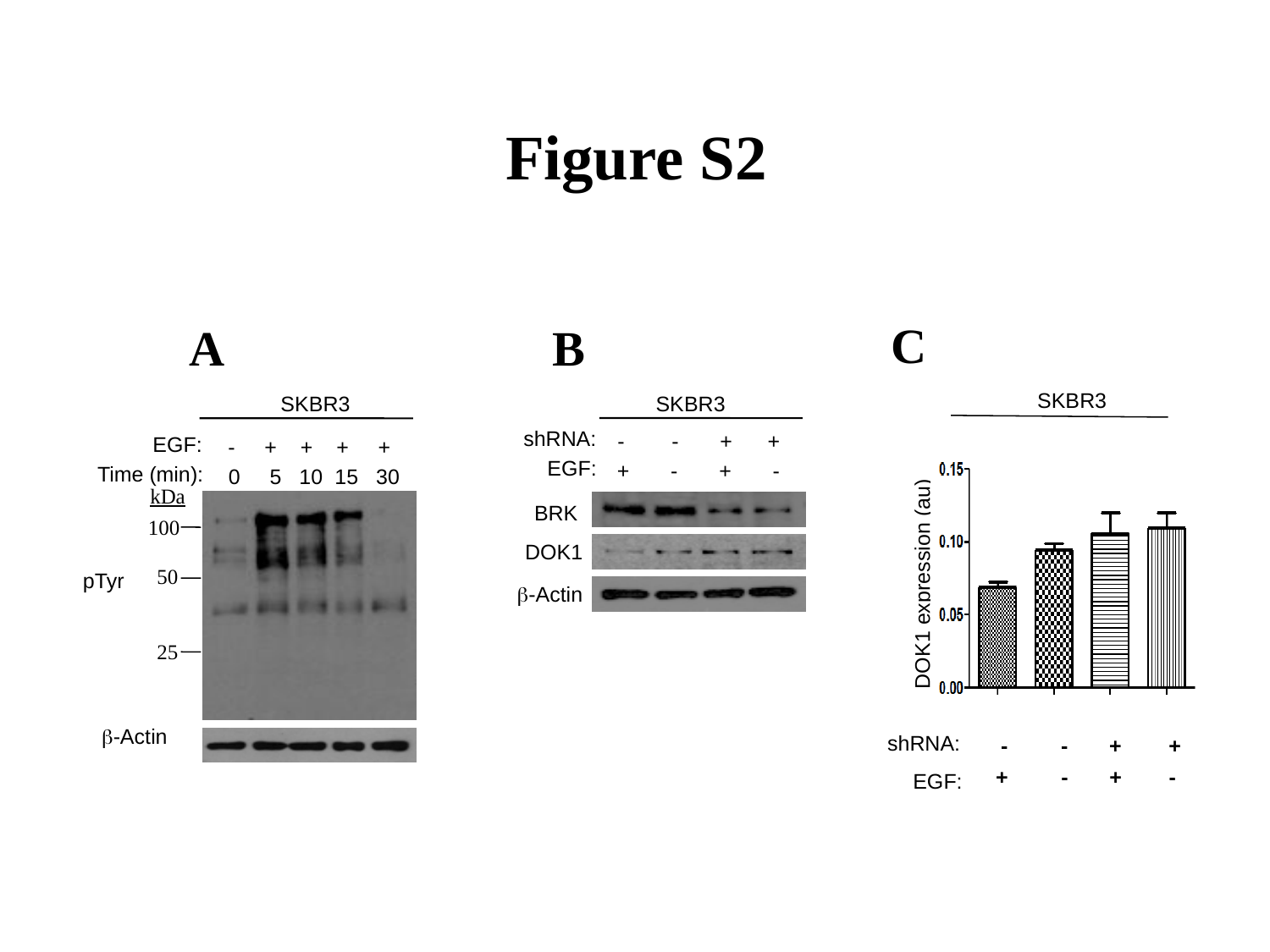

Figure S2
C
A
B
SKBR3
DOK1 expression (au)
shRNA:
- - + +
+ - + -
EGF:
SKBR3
shRNA:
 - - + +
EGF:
+ - + -
BRK
DOK1
β-Actin
SKBR3
EGF:
 - + + + +
Time (min):
 0 5 10 15 30
kDa
100
50
pTyr
25
β-Actin

## Slide 4
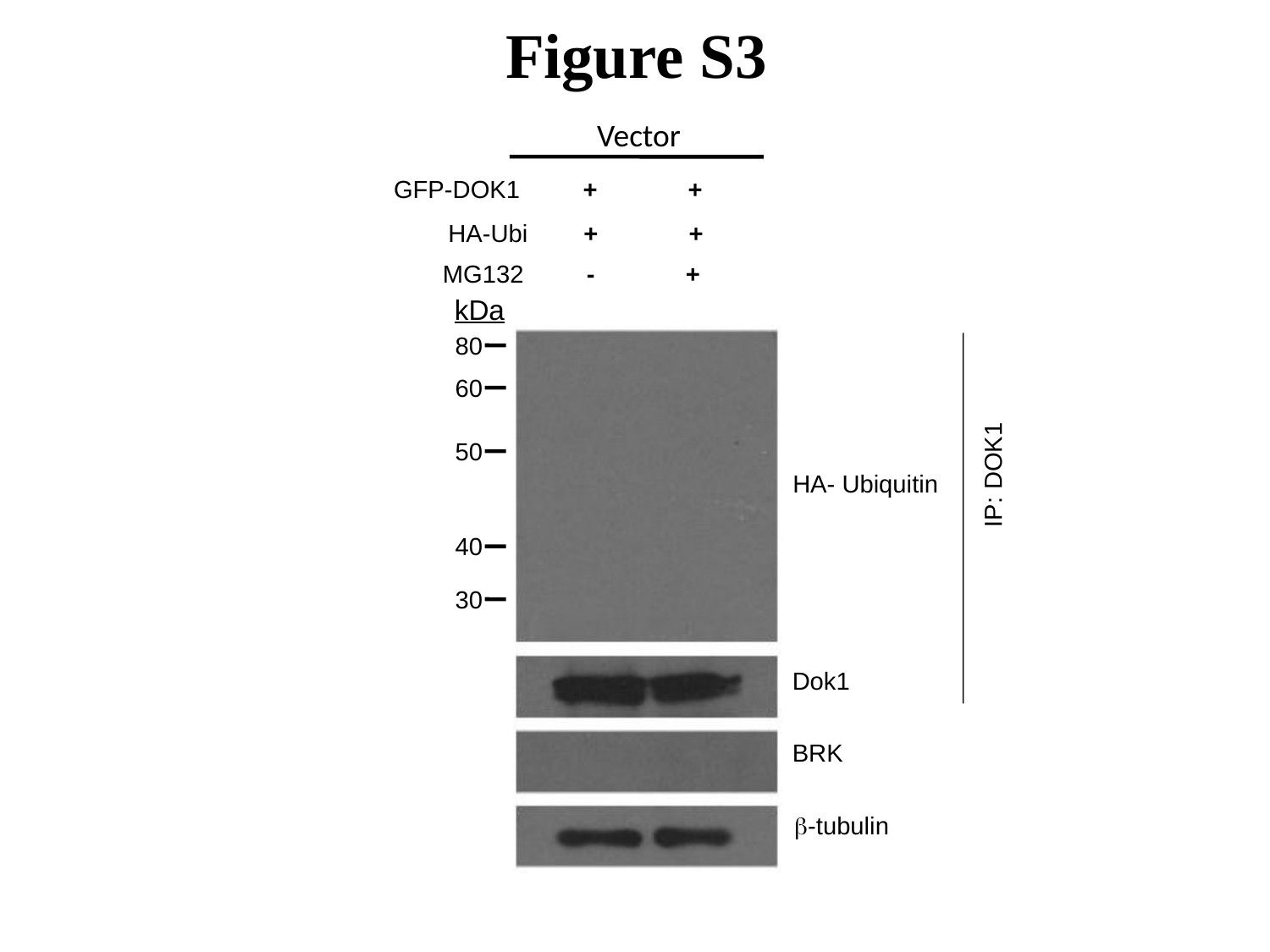

Figure S3
Vector
GFP-DOK1 + +
HA-Ubi + +
MG132 - +
kDa
80
60
50
IP: DOK1
HA- Ubiquitin
40
30
Dok1
BRK
β-tubulin

## Slide 5
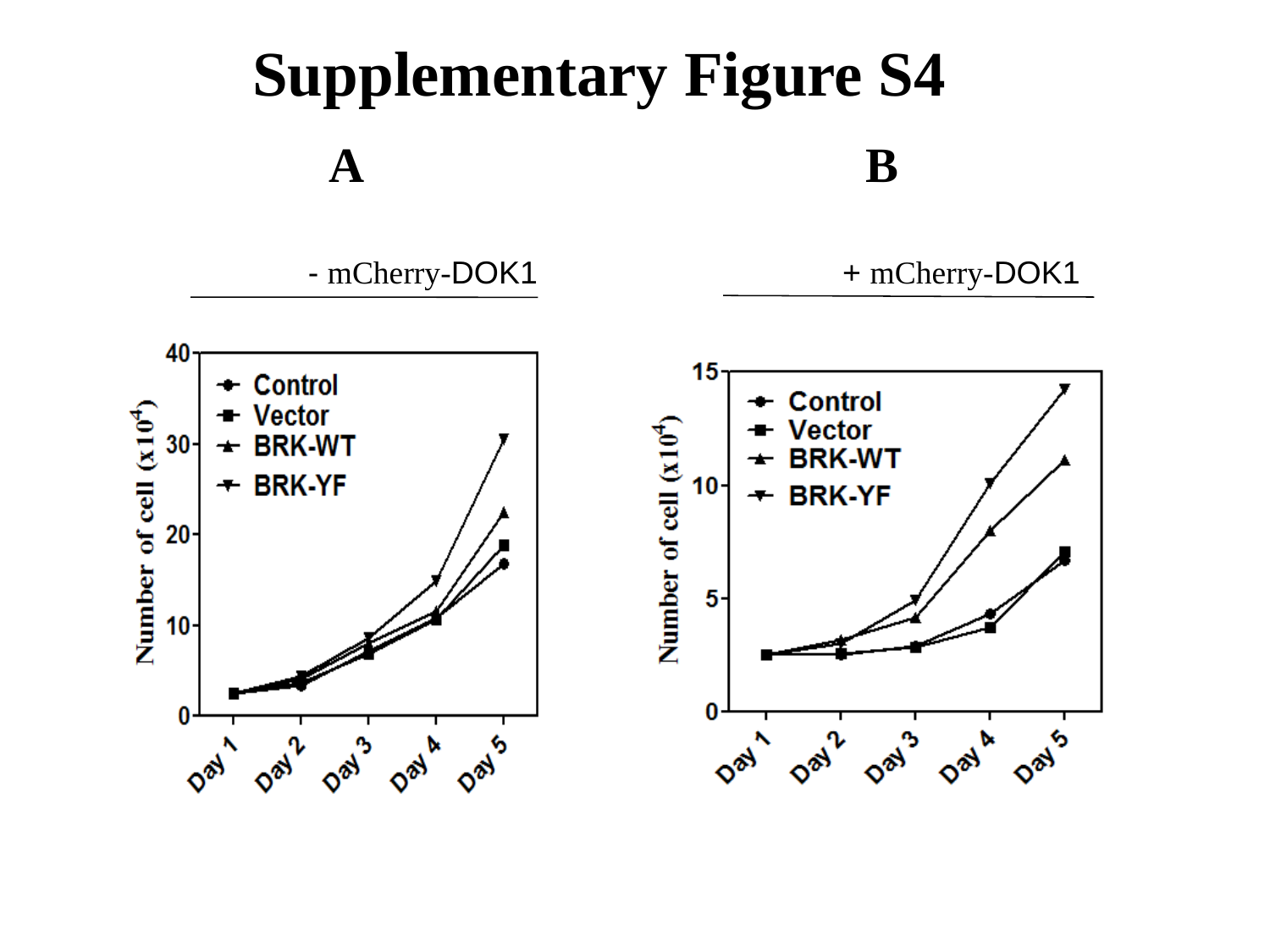

Supplementary Figure S4
A
B
- mCherry-DOK1
+ mCherry-DOK1

## Slide 6
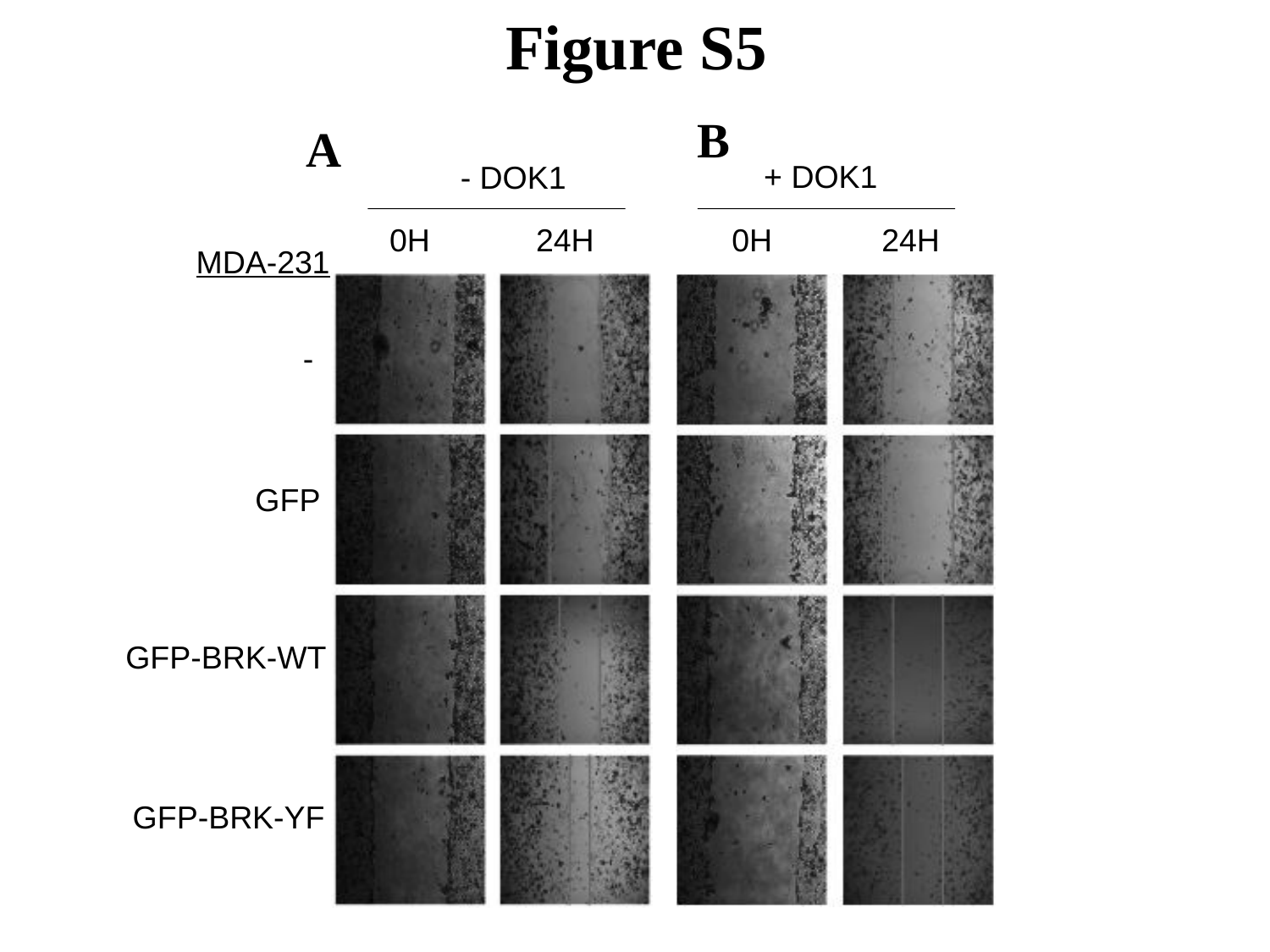

Figure S5
B
A
+ DOK1
- DOK1
0H
24H
0H
24H
MDA-231
-
GFP
GFP-BRK-WT
GFP-BRK-YF

## Slide 7
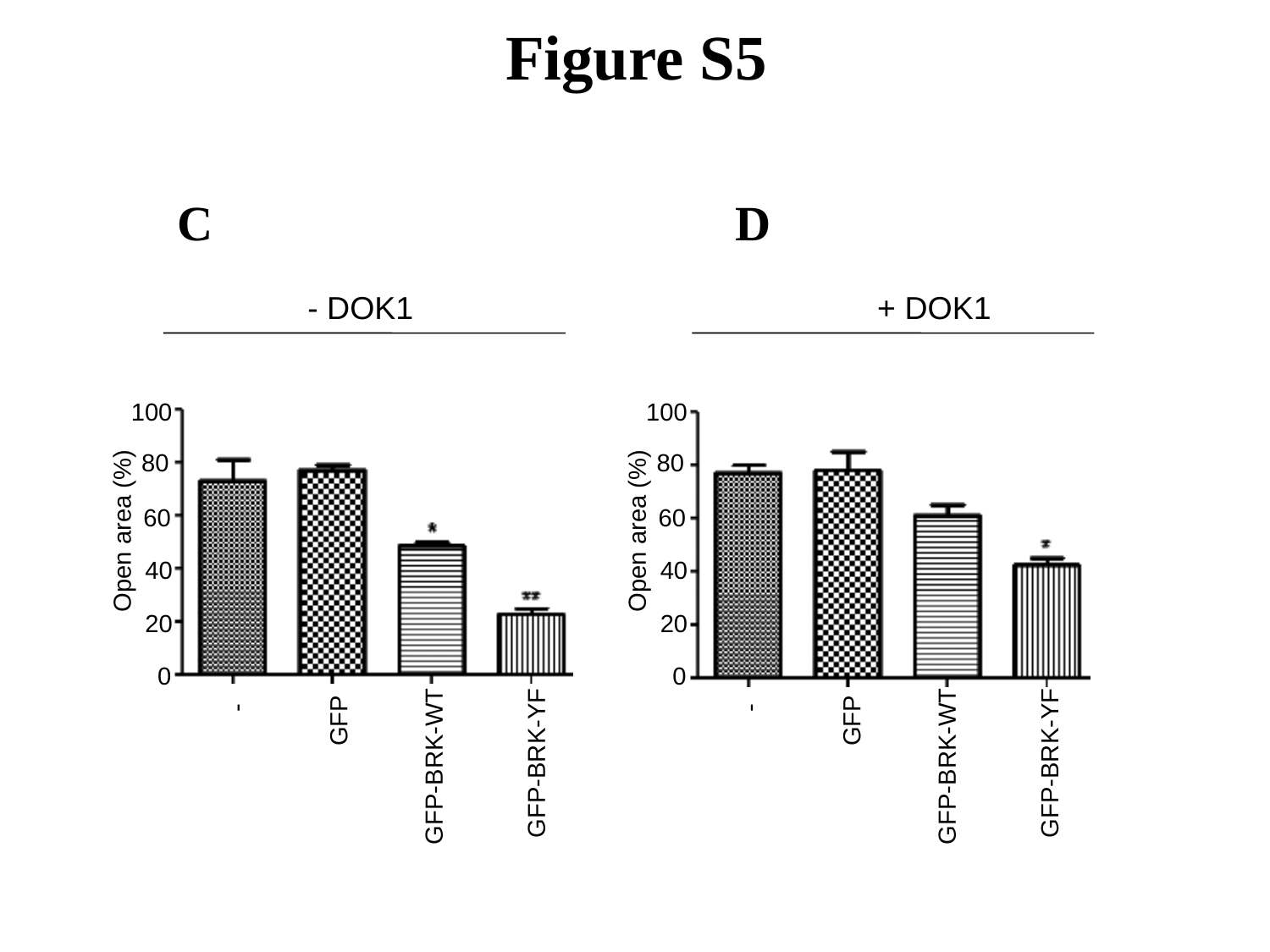

Figure S5
C
D
- DOK1
+ DOK1
100
100
80
80
60
60
Open area (%)
Open area (%)
40
40
20
20
0
0
-
-
GFP
GFP
GFP-BRK-YF
GFP-BRK-YF
GFP-BRK-WT
GFP-BRK-WT
